# Structural insights into human TFIIIC promoter recognition

**DOI:** 10.1126/sciadv.adh2019

**Published:** 2023-07-07

**Authors:** Wolfram Seifert-Davila, Mathias Girbig, Luis Hauptmann, Thomas Hoffmann, Sebastian Eustermann, Christoph W. Müller

**Affiliations:** ^1^ Structural and Computational Biology Unit, European Molecular Biology Laboratory (EMBL), Meyerhofstrasse 1, 69117 Heidelberg, Germany.; ^2^ Candidate for joint PhD degree from EMBL and Faculty of Biosciences, Heidelberg University, 69120 Heidelberg, Germany.

## Abstract

Transcription factor (TF) IIIC recruits RNA polymerase (Pol) III to most of its target genes. Recognition of intragenic A- and B-box motifs in transfer RNA (tRNA) genes by TFIIIC modules τA and τB is the first critical step for tRNA synthesis but is mechanistically poorly understood. Here, we report cryo–electron microscopy structures of the six-subunit human TFIIIC complex unbound and bound to a tRNA gene. The τB module recognizes the B-box via DNA shape and sequence readout through the assembly of multiple winged-helix domains. TFIIIC220 forms an integral part of both τA and τB connecting the two subcomplexes via a ~550–amino acid residue flexible linker. Our data provide a structural mechanism by which high-affinity B-box recognition anchors TFIIIC to promoter DNA and permits scanning for low-affinity A-boxes and TFIIIB for Pol III activation.

## Teaser

Cryo-EM reveals how human transcription factor IIIC binds tRNA genes with high affinity using a multi-step DNA binding mechanism.

## INTRODUCTION

Nuclear genes are transcribed by DNA-dependent RNA polymerases, which are recruited to promoter sequences by general transcription factors (GTFs) (*1*). Transcription factor (TF) IIIC, a six-subunit protein complex with a molecular weight (MW) of 624 kDa in humans, facilitates transcription of type 1 (5*S* ribosomal RNA) and type 2 [e.g., transfer RNAs (tRNAs)] RNA polymerase (Pol) III target genes (*2*–*5*). Whereas type 1 genes also require TFIIIA, TFIIIC is the first GTF that binds type 2 genes and subsequently recruits the three-subunit TFIIIB complex, composed of B-related factor 1 (BRF1), B double prime 1 (BDP1), and TATA-binding protein. TFIIIB binds upstream of the transcription start site and aids Pol III to bind and open the promoter DNA (*6*). TFIIIC is composed of two subcomplexes, τA and τB, that bind two gene internal DNA motifs: A-box and B-box (*7*, *8*). τA binds the A-box with low affinity and is required to recruit TFIIIB to the promoter DNA (*6*, *9*). τB interacts with the B-box with high affinity and, therefore, is essential for target DNA recognition of type 2 genes (*9*).

Deregulation of the Pol III transcription apparatus was reported in various cancers [reviewed in (*10*)], and increased expression and protein levels of TFIIIC subunits were found in ovarian cancers (*11*) and during tumoral transformation (*12*). Independently of its role in recruiting Pol III to its target genes, TFIIIC recognizes “extra TFIIIC” (ETC) sites in various eukaryotes (*13*–*15*) and functions in genome organization by acting as a chromatin insulator (*16*, *17*) and via interaction with the architectural proteins cohesin and condensin (*18*–*21*). TFIIIC also has histone acetylation activity and, together with the insulator protein CCCTC-binding factor, was shown to mediate long-range chromatin looping (*22*, *23*).

The yeast TFIIIC complex is composed of the subunits τ131, τ95, and τ55, forming τA, and of τ138, τ91, and τ60, forming τB (*24*). For human TFIIIC (hTFIIIC), only five subunits with MWs of 230, 110, 102, 90, and 63 kDa were initially shown to form a stable functional complex, termed TFIIIC2 (*25*). TFIIIC2 was identified together with another functional entity, termed TFIIIC1, that was required to facilitate gene transcription (*26*) and could later on be shown to contain human BDP1 (*27*). The sixth hTFIIIC subunit, TFIIIC35, was later identified via sensitive sequence homology detection, molecular cloning, and biochemical characterization (*28*).

TFIIIC must have a high degree of structural elasticity because A- and B-box binding implies spanning the entire tRNA gene body (*29*, *30*), whose size varies from 70 to 108 base pairs (bp) in *Homo sapiens* and 71 to 133 bp in *Saccharomyces cerevisiae*. Owing to this structural elasticity, our molecular understanding of how TFIIIC is organized has been limited to structures of subassemblies of τA and τB (*31*–*35*). Structural studies of the τA subcomplex showed that τ95 and τ55 form a heterodimer (*33*), whereas τ131 features a tetratricopeptide repeat (TPR) array (*34*). The τ91 and τ60 subunits both contain a WD40 repeat domain with a β-propeller structure that interact with each other to form a rigid τB core (*31*). The largest TFIIIC subunit, τ138, was predicted to contain multiple winged-helix (WH) domains, of which one was crystallized (*34*). The structural insights from the TFIIIC subassemblies were further combined with cross-linking mass spectrometry to propose a model of the overall TFIIIC complex (*34*), and a recent cryo–electron microscopy (cryo-EM) study revealed the overall structure of the τA subcomplex (*35*).

Besides the in-depth molecular investigation of the τA and τB subcomplexes, it remained enigmatic how τA and τB interact with each other and, thereby, form a stable and, at the same time, flexible molecular assembly. Furthermore, molecular insights into how TFIIIC recognizes its target genes were missing because no structures of DNA-bound TFIIIC have been experimentally determined. In addition, structural insights into the hTFIIIC complex are lacking, which limits our understanding of the human Pol III transcription machinery.

## RESULTS

### Cryo-EM reveals the molecular structures of human τA and τB

**Fig. 1.**
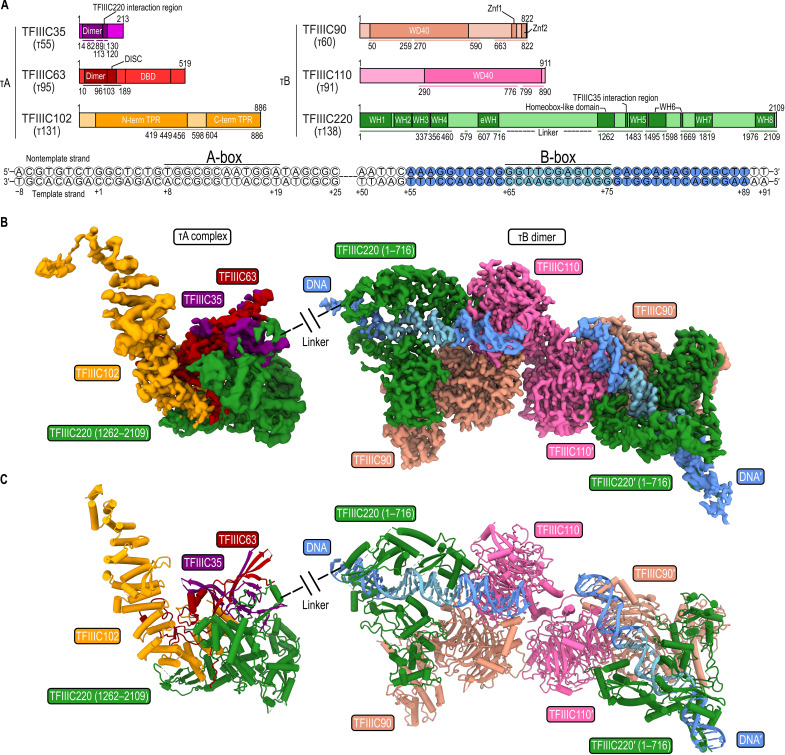
Cryo-EM structure of hTFIIIC. (**A**) Domain diagram of hTFIIIC subunits (top) and the tRNA gene used for cryo-EM analysis (bottom). Nucleotides are numbered according to the start of the predicted mature tRNA. Yeast homologs are given in parentheses. Colored bars, built regions; colored circles, modeled DNA. DBD, DNA binding domain; TPR, tetratricopeptide repeat domain; WD40, WD40 repeat domain; WH, winged-helix domain; eWH, extended winged-helix domain. (**B**) Cryo-EM maps of human τA and dimeric, DNA-bound τB. (**C**) Atomic models of human τA and DNA-bound τB.

hTFIIIC consists of τA subunits TFIIIC35, TFIIIC63, and TFIIIC102 and τB subunits TFIIIC90, TFIIIC110, and TFIIIC220 (Fig. 1A). To obtain structural insights into hTFIIIC, we coexpressed its subunits in insect cells and confirmed by mass photometry (MP) that the purified sample corresponds to the expected size of the intact hTFIIIC complex (MW_observed_ = 654 ± 52 kDa, MW_expected_ = 624 kDa) (fig. S1, A to C). Reconstitution with a type II promoter DNA encoding the human *TRR-TCT3-2* tRNA gene (MW, 61 kDa) gave rise to a complex of 701 ± 46 kDa, indicating cognate DNA binding activity of the complex at low nanomolar concentrations, which we confirmed with a filter binding assay (fig. S1, D and E). DNA binding was furthermore demonstrated with an electrophoretic mobility shift assay (EMSA) (fig. S1F).

We determined cryo-EM structures of hTFIIIC alone and in complex with the *TRR-TCT3-2* tRNA gene. In both cases, we obtained two distinct sets of particles for human τA and τB. Separate cryo-EM reconstructions at 3.5- and 3.2-Å resolution (DNA-bound) (fig. S2) and at 3.8- and 3.4-Å resolution (DNA-unbound) (fig. S3) enabled us to build and refine atomic models of human τA and τB, respectively (Fig. 1, B and C; fig. S4, A to D; and table S1).

Unexpectedly, we observed a dimeric structure of τB in both the DNA-unbound and DNA-bound three-dimensional (3D) reconstructions (fig. S5A). The two τB subcomplexes are held together by apolar and polar interactions between two copies of subunit TFIIIC110 (fig. S5B). TFIIIC dimerization is likely to be concentration dependent because our MP measurements did not show clear indications for dimer formation at nanomolar concentrations. The observation that TFIIIC dimerizes under cryo-EM conditions should be treated with care because we were unable to evaluate TFIIIC110 dimerization in solution due to a limited amount of protein sample. Because TFIIIC also functions in 3D genome organization (*22*, *23*), one could, nevertheless, speculate that DNA-bound τB dimerizes at tRNA gene clusters and ETC sites, where it may help to stabilize long-range chromatin loops. For the following molecular description of human τB, we will, however, only focus on the τB monomer.

### The human τB core structure stabilizes two WH domains of TFIIIC220

**Fig. 2.**
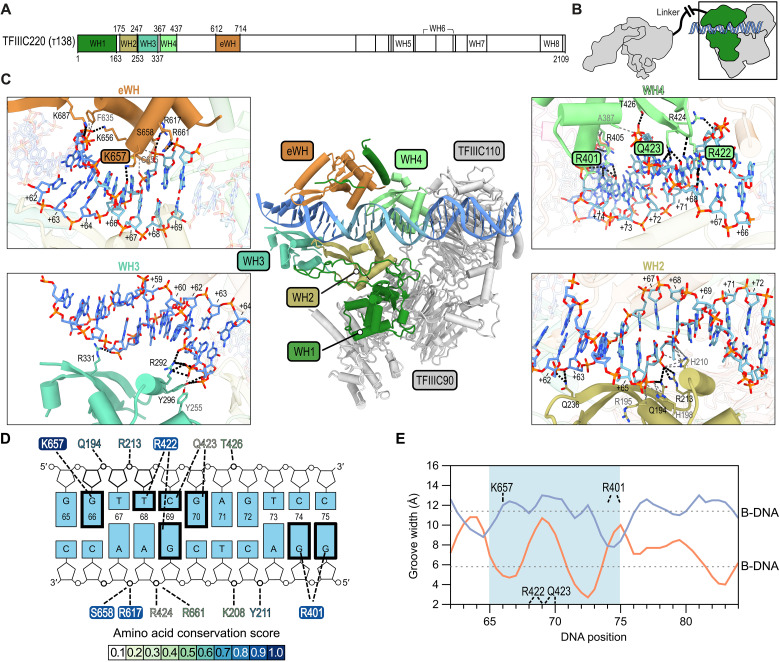
Interaction of τB with B-box promoter DNA. (**A**) Domain architecture of TFIIIC220. N-terminal DBDs are colored. (**B**) Schematic of hTFIIIC bound to DNA. Green, TFIIIC220 N-terminal moiety. (**C**) Centre: Atomic model of human τB bound to DNA (light blue, B-box). Contacts between WH2, WH3, WH4, and eWH with the DNA are shown as close-up views. Black dashed lines, hydrogen (H) bonds; gray dashed lines, apolar contacts. H-bond–forming residues are labeled, and amino acids that bind the DNA-bases are highlighted in colored boxes. (**D**) Nucleotide plot of the polar B-box DNA-TFIIIC220 interaction. H-bonds are depicted as bold, dashed lines. Bases that are contacted directly are highlighted as bold frames. Contacting residues are colored according to their Scorecons (*77*) conservations scores. Residues with a conservation score > 0.9 are framed. (**E**) DNA groove width analysis of the TFIIIC220-bound DNA, computed with the Curves+ software (*67*). The B-box motif is highlighted in light blue. Orange and blue curves show DNA minor and major groove widths, respectively. Dotted lines represent minor and major groove widths of ideal B-DNA. TFIIIC220 residues that form base-specific contacts are depicted above the nucleotide positions.

The core of human τB is formed by TFIIIC90 and TFIIIC110 (τ60 and τ90 in yeast), which stably associate with each other via their WD40 domains (fig. S6A). We could also assign the N-terminal third (1 to 716) of subunit TFIIIC220 (τ138 in yeast) featuring four WH domains and one extended WH (eWH) domain to human τB (Figs. 1B and 2). TFIIIC220-WH1 (1 to 163) engages with the WH2 domain (175 to 247) (fig. S6B) and binds two zinc fingers of the TFIIIC90 C-terminal domain (fig. S6C). On the other edge of τB, the TFIIIC110-WD40 domain stabilizes the TFIIIC220-WH4 (367 to 437) domain.

Hence, TFIIIC90-TFIIIC110 form, with the help of the TFIIIC220 WH1 domain and the C-terminal zinc fingers in TFIIIC90, a stable scaffold, which stabilizes the WH2 and WH4 domains. These two domains were, therefore, already visible in the absence of DNA, whereas the WH3 and eWH domains could not be visualized in the DNA-unbound human τB cryo-EM map (fig. S5A).

### TFIIIC recognizes the B-box motif via DNA shape and sequence readout

The cryo-EM structure of DNA-bound human τB enabled us to unveil the molecular basis of B-box DNA recognition. The 3.2-Å cryo-EM map of DNA-bound τB was of sufficient quality to assign 35 bp (55 to 89) of the DNA sequence (Fig. 1A). Upon DNA binding, the TFIIIC220-WH3 (253 to 337) and the eWH-domains (612 to 714) become ordered and facilitate B-box (65 to 75) DNA recognition together with WH2 and WH4 (Fig. 2 and fig. S5A). The DNA is, downstream of the B-box, additionally stabilized by a positively charged surface of TFIIIC110 (fig. S6D). The lack of this subunit in human has been associated with a transcriptionally inactive TFIIIC form (*36*). In yeast, the subunit τ91 (TFIIIC110 in human) has been shown to cooperate with τ138 (TFIIIC220 in human) for DNA binding (*37*). The WH domains huddle against the DNA from both sides and form several apolar and polar contacts with the phosphate backbone and bases of the B-box DNA (Fig. 2D and fig. S7A). By analyzing the B-box DNA sequence conservation, we found that the DNA bases that are contacted in a base-specific manner (G66, T68, C69, and G70 on the nontemplate strand and G69, G74, and G75 on the template strand) are conserved not only in human tRNA genes but also across a wide range of eukaryotes (fig. S7B). In TFIIIC220, the basic amino acids (R401, R422, and K657) that form base-specific contacts with the B-box DNA are also highly conserved (Fig. 2D, figs. S8 and S9, and table S3). Notably, biochemical DNA binding studies with yeast TFIIIC and the *SUP4* gene have shown that the mutations C56➔G and G57➔C (corresponding to C69 and G70 on the nontemplate strand in the human TRR-TCT3-2 tRNA gene) caused a 370- and 56-fold decrease, respectively, in DNA binding affinity (*38*). Our structural analysis, together with these previously conducted experiments, thus, confirms the importance of the recognition of these conserved bases. The geometry of the DNA is also substantially altered nearby the bases that appear to be contacted in a conserved manner (Fig. 2E). K657 (eWH) binding induces the opening of the DNA major groove and compaction of the corresponding minor groove, whereas R422/Q423 and R401 (WH3) widen the DNA minor groove. Hence, TFIIIC initiates recognition of the tRNA gene promoter in an evolutionary conserved manner via a combined DNA shape and sequence readout of the B-box DNA motif.

### TFIIIC220 connects τA and τB with a flexible linker

**Fig. 3.**
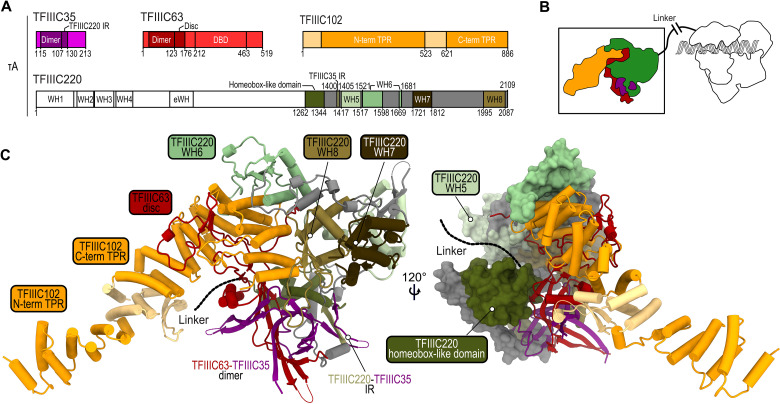
The C-terminal region of TFIIIC220 is an integral part of the τA subcomplex. (**A**) Diagram of the protein domains (colored) that form a stable τA subcomplex. For TFIII220, unmodeled regions are colored gray. IR, interaction region. (**B**) Schematic representation of τA (colored) and τB (white) connected by a flexible linker. (**C**) Atomic model of τA showing the C terminus of TFIIIC220 as cartoon (left) and as surface (right). The flexible linker connecting τA and τB is shown as black dashed lines.

Our cryo-EM analysis yielded a high-resolution structure of human τA, which, however, remained in the DNA-unbound state in the presence of the tRNA gene (Fig. 3). This observation is in agreement with biochemical studies on hTFIIIC, where the TFIIIC2 component (*25*, *36*, *39*) interacts exclusively with B-box and is deficient in interacting with A-box (*26*, *40*, *41*). However, upon the addition of the TFIIIC1 component, in which full composition remains unclear but has human BDP1 (*27*), the protection against deoxyribonuclease (DNase) I expands to the A-Box and the transcription start site. On the other hand, in yeast TFIIIC, it has been shown that τA binds the DNA with low affinity and in an unspecific manner (*9*, *24*, *32*).

When inspecting the 3D reconstruction of human τA, we found that 50% of the cryo-EM density corresponds to the τA subunits TFIIIC35, TFIIIC63, and TFIIIC102 (τ55, τ95, and τ131 in yeast) (Fig. 3A). TFIIIC35-TFIIIC63 form a heterodimer that binds the C-terminal TPR domain of TFIIIC102 (Fig. 3C). Human τA, thus, partly resembles its yeast counterpart, but it lacks the phosphatase domain of τ55 (absent in hTFIIIC35) and the cryo-EM density for the TFIIIC63–DNA binding domain (DBD). Unexpectedly, the remaining ~50% of the cryo-EM density belongs to the C-terminal half of the τB subunit TFIIIC220 (Fig. 3). The corresponding atomic model features a homeobox-like domain, interacting with the TFIIIC35-TFIIIC63 heterodimer, and four WH domains (WH5 to WH8) (fig. S10A), of which WH6 and WH8 directly contact TFIIIC102. TFIIIC220, thereby, partly occupies the same binding interface of TFIIIC102 as the TFIIIC63-DBD in yeast (fig. S11).

The C-terminal half of TFIIIC220 forms an integral part of human τA, suggesting that TFIIIC220 links τA and τB with a ~550-residue-long linker between its N and C termini. To address this hypothesis, we used our cryo-EM data to measure the distance between those particles that contribute initially to the τA and τB reconstructions (see Materials and Methods). Our measurement revealed a distinct distribution of particle distances, peaking at 200 Å, which differs from a set of simulated, randomly distributed particles (fig. S12, A to C), indicating that the τA particles are stably and, at the same time, flexibly linked to the tRNA gene-bound τB fraction. This flexibility allows TFIIIC to bridge the variable distances between A-box and B-box present in different tRNA genes in the human genome (fig. S12D). Notably, human TFIIIC220 is, with 2109 amino acids, substantially larger than its *S. cerevisiae* ortholog, τ138 (1161 amino acids) (fig. S10B). Nevertheless, τ138 is predicted to contain, with exception of the WH8 domain, the same number of WH domains that are also arranged in the same order as in human TFIIIC220, suggesting that the overall arrangement of TFIIIC is conserved. With only ca. 100 amino acids, the linker between the τ138 N and C termini is shorter than its human counterpart, but, given its flexible nature, we assume that this length is sufficient to also bridge the yeast τA and τB subcomplexes and allows simultaneous A- and B-box binding on yeast tRNA genes.

## DISCUSSION

**Fig. 4.**
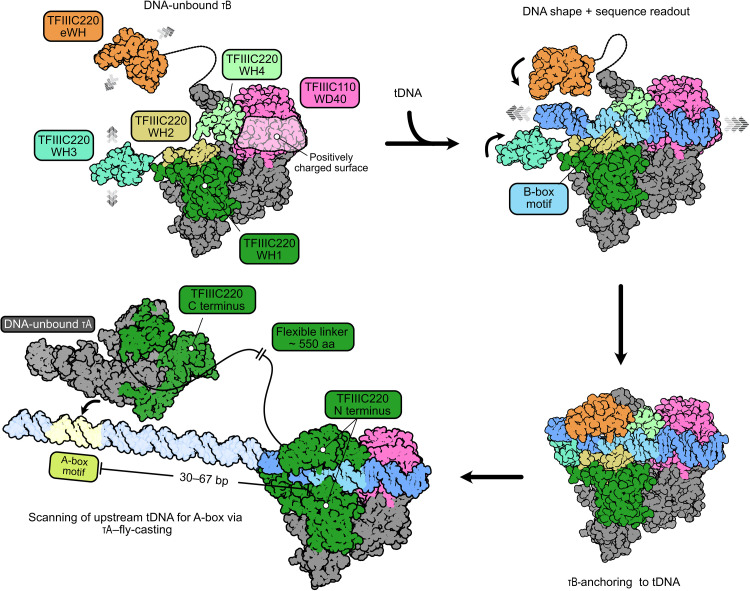
Model of TFIIIC promoter recognition. TFIIIC110 and TFIIIC90 along with the TFIIIC220-WH1, WH2, and WH4 domains form a rigid τB scaffold, while WH3 and eWH domains remain flexible. The DNA is initially recognized by the WH2 and WH4 domains and, subsequently, by the WH3 and eWH domains via shape and sequence readout. In addition, the TFIIIC110-WD40 positively charged pocket (transparent-white surface) helps to stabilize the interaction with the DNA molecule. Once TFIIIC is anchored to the tRNA gene, the τA subcomplex, connected by a ~550–amino acid (aa) flexible linker to τB, will search for the A-box motif, separated from the B-box with a variable distance between 30 and 67 bp, by a fly-casting mechanism.

On the basis of our structural analysis, we propose a multistep mechanism of tRNA gene promoter recognition via TFIIIC (Fig. 4). The first step involves the high-affinity DNA sequence and shape readout of the B-box motif via an assembly cascade of WH domains. WH2 and WH4 of TFIIIC220 are an integral part of the rigid τB-core, formed by TFIIIC90-TFIIIC110 and the TFIIIC220-WH1 domain, and their conformation does not change upon DNA binding, suggesting that they provide the platform for initial DNA recognition. We speculate that τB-WH2-WH4 mediates scanning of the tRNA gene for the correct B-box motif. In addition, the TFIIIC-DNA interaction is further stabilized by the WD40 domain in TFIIIC110, which binds the DNA downstream of the B-box via a positively charged groove. Subsequently, the WH3 and eWH domains recognize the upstream half of the B-box, and the WH domains, collectively, enclose the B-box motif and its upstream region.

The concerted engagement of the B-box motif by τB WH domains at up- and downstream sites anchors TFIIIC to the tRNA gene and allows, via the flexible TFIIIC220 linker of 550 residues connecting τA and τB, subsequent scanning of the tRNA gene for low-affinity A-box motifs through a fly-casting mechanism. Composed of, in total, nine WH domains, TFIIIC220 fulfills multiple key roles for TFIIIC-mediated promoter recognition. Whereas the N-terminal WH1 to WH4 and eWH domains either directly or indirectly (WH1) mediate B-box recognition, the C-terminal WH5 to WH8 domains form an integral part of τA and, thereby, serve as a hook that tethers τA and τB. The flexible TFIIIC220 linker between τA and τB also equips TFIIIC with the required elasticity to adapt to the variable distances between the A- and B-box motifs found across different tRNA genes.

It remains to be determined how TFIIIC recognizes the A-box motif and recruits TFIIIB. A potential reason why we captured human τA exclusively in the DNA-unbound state could be due to posttranslational modifications that may regulate the affinity of τA to the A-box motif. An alternative explanation is that human τA mainly did not bind the A-box due to the absence of TFIIIB. TFIIIC functions as an assembly factor for TFIIIB (*6*), but TFIIIB, vice versa, enhances the protection of the A-box DNA region in DNase I footprint experiments in yeast (*42*), suggesting that TFIIIB and τA cooperatively recognize the promoter DNA. Yeast TFIIIB and TFIIIC interact with each other in solution via the TFIIIB subunit BRF1 and the τA subunit τ131 (TFIIIC102 in humans) (*35*, *43*–*45*). It might, thus, be plausible that B-box–mediated anchoring of TFIIIC also allows τA to “fish” for BRF1 in solution, thereby promoting TFIIIB assembly upstream of the transcription start site, which could, in turn, stabilize the association between τA and the A-box.

## MATERIALS AND METHODS

### Protein expression

The six hTFIIIC-coding genes, codon-optimized for expression in insect cells, were ordered from DNASU (*46*). Two genes were edited via polymerase chain reaction (PCR) to match the protein sequences found on UniProt (*47*). The original *GTF3C2* gene encoded 12 additional amino acids, and *GTF3C3* encoded a serine in position 70 instead of asparagine. In addition, *GTF3C6* encoded a serine in position 1, which was replaced by methionine. A restriction-free cloning protocol was applied to clone all the TFIIIC genes into a pACEBac vector. Using an overlap extension PCR cloning, a tobacco etch virus–cleavable His-tag was added to the N terminus of *GTF3C2* and *GTC3C5*, followed by the addition of a 3× FLAG-tag to the C terminus of *GTF3C1*. A six-gene TFIIIC expression plasmid was cloned via biGBac (*48*). The genes belonging to τA and τB were cloned into the pBIG1a and pBIG1b plasmids, respectively, and the subcomplex-expression cassettes were cloned into pBIG2ab. A recombinant baculovirus was constructed following the standard procedures.

Sf21 insect cells were used to express the TFIIIC complex. Sf21 cells were diluted to 0.5 × 10^6^ cells/ml and immediately infected with the TFIIIC-containing baculovirus. Sf21 cells were grown up to 72 hours at 27°C or until their cell viability reached 90 to 95%. Cells were harvested by centrifugation at 700*g*, followed by a washing step with phosphate-buffered saline, flash-frozen, and stored at −80°C until use.

### Protein purification

Frozen pellets were resuspended for 2 hours at 4°C while stirring gently in 3× lysis buffer [20 mM Hepes (pH 7.5), 0.25 mM dithiothreitol (DTT), 500 mM NaCl, 2 mM MgCl_2_, 0.1% NP-40, 10% glycerol, and 0.25 mM DTT], supplemented with 1:1000 DNase I, SigmaFast EDTA-free protease inhibitors tablet per 20 g of cell pellet, 4 μl of benzonase per 50 ml of lysis buffer. A lysis step to the resuspended pellet using a sonicator for 3 min was applied. The lysate was centrifuged at 30,000*g* for 1 hour at 4°C to remove cell debris. Anti-FLAG M2 agarose beads (Sigma-Aldrich) were incubated with the remaining supernatant for 2 hours on a rolling plate at 4°C. Beads were loaded onto an Econo-Column Chromatography column and washed with 30× column volumes (CV) of lysis buffer, followed by 25 CV of wash buffer 1 [20 mM Hepes (pH 7.5), 500 mM NaCl, 10% glycerol, 0.1% NP-40, and 0.25 mM DTT]. The washed resin was incubated with 2× CV of elution buffer 1 [20 mM Hepes (pH 7.5), 500 mM NaCl, 10% glycerol, 0.1% NP-40, 0.25 mM DTT, and FLAG peptide (0.2 mg/ml)] for 30 min at 4°C. Wash buffer 2 [20 mM Hepes (pH 7.5), 100 mM NaCl, 10% glycerol, 0.1% NP-40, and 0.25 mM DTT] was added to the eluted sample to dilute the initial salt concentration to 200 mM NaCl and then applied to a Capto HiRes Q 5/50 (Cytiva) column pre-equilibrated in buffer A [20 mM Hepes (pH 7.5), 200 mM NaCl, 5% glycerol, and 5 mM DTT]. Sample elution was carried out by applying a linear gradient from 0 to 100% buffer B [20 mM Hepes (pH 7.5), 1 M NaCl, 5% glycerol, and 5 mM DTT] over 70 ml. Peak fractions were analyzed by SDS–polyacrylamide gel electrophoresis. The fractions containing TFIIIC were pooled, concentrated up to 2 mg/ml, buffer-exchanged using storage buffer [20 mM Hepes (pH 7.5), 200 mM NaCl, and 5 mM DTT], flash-frozen, and stored at −80°C until use or directly used for cryo-EM sample preparation.

### DNA oligonucleotide preparation

The human *TRR-TCT3-2* gene (5-acgtgtctGGCTCTGTGGCGCAATGGATAGCGCATTGGACTTCTAGATAGTTAGAGAAATTCAAAGGTTGTG**GGTTCGAGTCC**CACAGAGTCG*C*tttt-3, the A-box is underlined, and the B-box is printed in bold, and nucleotides flanking the mature tRNA product are depicted as lower case) was ordered from Sigma-Aldrich. Nontemplate and template strands were annealed in H_2_O followed by heating to 95°C for 5 min before cooling down to 20°C at a rate of 1°C/min. The DNA mixture was applied to a Superdex 200 increase 3.2/300 (Cytiva) column pre-equilibrated in DNA buffer [20 mM Hepes (pH 7.5), 150 mM NaCl, 5 mM MgCl_2_, and 5 mM DTT] to remove incomplete products and impurities. The fractions corresponding to the major peak were pooled and quantified by nanodrop. The sample was stored at −20°C until use.

### MP sample preparation, data acquisition, and processing

High-precision microscope coverslips (24 mm by 50 mm) were prepared in the following manner: (i) wash with ddH_2_O; (ii) wash with isopropanol; (iii) wash with ddH_2_O; (iv) wash with isopropanol; (v) rinse with ddH_2_O; and (vi) dry under a pressurized air stream. A silicone gasket with six cavities was placed on top/center of the coverslip to form reaction holes. A total amount of 19 μl of working buffer 1 [20 mM Hepes (pH 8), 150 mM KCl, 2 mM MgCl_2_, and 5 mM DTT] for hTFIIIC alone and working buffer 2 [20 mM Hepes (pH 8), 200 mM KCl, 2 mM MgCl_2_, and 5 mM DTT] for hTFIIIC-DNA sample was applied to each reaction hole for autofocus stabilization before every measurement. One microliter of the sample at an initial concentration of 400 nM was added to the working buffer 1 or 2. All measurements were performed using a Refeyn Two^MP^ mass photometer (Refeyn Ltd., Oxford, UK). Videos of 1 min with regular image size (150 × 59 binned pixels, 10.9 μm–by–4.3 μm imaged area, 46.3-μm^2^ detection area) were recorded using the Acquire^MP^ software (Refeyn Ltd., version 2.4.0), and data analysis was performed using the Discover^MP^ software (Refeyn Ltd., version 2.4.0). To generate the standard contrast-to-mass calibration curve [linear regression fit with coefficient of determination (*R*^2^) of 0.99999], bovine serum albumin (66-kDa) and immunoglobulin G (150- and 300-kDa) proteins, diluted in working buffer 1, were used.

### Electrophoretic mobility shift assay

The EMSA samples contained double-stranded human *TRR-TCT3-2* DNA oligonucleotides (prepared as described above) at a concentration of 0.2 μM, which were incubated with hTFIIIC in EM buffer [20 mM Hepes (pH 8), 225 mM KCl, 2 mM MgCl_2_, and 5 mM DTT] at room temperature (RT) for 10 min. A range of hTFIIIC protein concentrations (0.1, 0.2, 0.3, 0.4, and 0.8 μM) were used. To each sample, 50% glycerol prepared in EM buffer was added to reach a final concentration of 10%, and the resulting mixture was loaded onto a 0.8% agarose gel prepared in 0.5× tris-borate buffer [44.6 mM tris and 44.5 mM boric acid (pH 8.3)]. The electrophoresis was performed at 85 V for 45 min at 4°C in the same buffer used to prepare the agarose gel. Gels were imaged in a Quantum CX5 imaging system (Vilber).

### DNA labeling and filter binding assay

The template and nontemplate oligonucleotides of the human *TRR-TCT3-2* gene were labeled at the 5′ end with [γ-32P] adenosine 5′-triphosphate using T4 polynucleotide kinase (New England Biolabs). The labeled oligonucleotides were then loaded onto a 10% acrylamide/bisacrylamide gel containing 8.3 M (w/v) urea. After electrophoresis, the band corresponding to the full-length oligonucleotide was excised and eluted overnight in 0.5 M ammonium acetate, 10 mM magnesium acetate, 0.1% (w/v) SDS, and 0.1 mM EDTA buffer. The eluted oligonucleotide was then ethanol precipitated.

To perform the filter binding assay, the labeled oligonucleotide was annealed to its complementary strand in 20 mM Hepes (pH 7.5), 5 mM MgCl_2_, and 100 mM KCl for 30 min at RT. Fixed amount of radioactive DNA (~0.5 nM) was incubated with increasing amounts of TFIIIC (0.1 nM to 1 μM) in reconstitution buffer [20 mM tris-HCl (pH 7.5), 150 mM KCl, 2 mM MgCl_2_, and 5 mM DTT] for 30 min at 4°C. The DNA-protein mixture was then filtered through a 0.45-μm nitrocellulose filter (Whatman), and the filters were counted in a Tri-Carb 2800TR Cerenkov scintillation counter (PerkinElmer). The counts were normalized and fitted to a Hill equation with a fixed Hill coefficient of 1 using Prism (GraphPad).

### Cryo-EM sample preparation

To reconstitute the hTFIIIC-DNA complex for cryo-EM, freshly purified hTFIIIC at an initial concentration of 3.17 μM was incubated with human *TRR-TCT3-2* DNA (7.5 μM stock concentration) at a 1:1 molar ratio for 10 min at RT. The mixture was passed through a Zeba Spin desalting column previously equilibrated with EM buffer. The sample was left at RT for 30 min, then mixed 0.8:0.2 (v/v) with 0.5% (w/v) of octyl-β-glucoside (previously dissolved in EM buffer) to a final concentration of 0.1% (w/v/) octyl-β-glucoside, applied to freshly plasma-cleaned 200-mesh Au R2/2 grids (UltrAuFoil), and plunge-frozen in liquid ethane using a Vitrobot Mark IV (Thermo Fisher Scientific), which was set to 100% humidity and 6°C. To prepare the cryo-EM sample of DNA-unbound hTFIIIC, the same protocol was used without using the desalting column step.

### Cryo-EM data acquisition and processing

Cryo-EM data of the hTFIIIC-DNA sample were collected on a Titan Krios G3 (Thermo Fisher Scientific) operated at 300 keV, equipped with a Gatan K3 detector and energy filter. A magnification of ×105,000, corresponding to a physical 0.822 Å per pixel, was used. A total amount of 11,025 image stacks of 40 frames were collected in counting mode with a total electron dose of 42.8 electrons/Å^2^ at defocus range from 0.7 to 1.7 μm using SerialEM (*49*). The dataset of DNA-unbound hTFIIIC (11,063 micrographs) was collected with the same imaging parameters (tables S1 and S2). The data processing steps for this dataset are illustrated in fig. S3. To avoid redundancy, we specially focus on the DNA-bound TFIIIC sample and describe its data processing below.

Micrographs were initially preprocessed using WARP (*50*). Particle coordinates were obtained by using the BoxNet2_20180918 model (without retraining) implemented in WARP. Subsequently, micrographs were preprocessed in RELION 3.1.3 (*51*) by using its implementation of MotionCor2 (*52*). Contrast transfer function (CTF) parameters were derived using Gctf (*53*). An initial set of 530,171 particles identified by WARP was extracted with a box size of 480 pixels and imported into cryoSPARC 3.3.2 (*54*) for all subsequent classification and refinement steps. 2D classification identified two sets of particles that correspond to τA and τB-DNA subcomplexes. Each set of particles was used to train neural networks (conv127 model) for particle picking in TOPAZ (*55*). For τA, a first round of particle picking in TOPAZ yielded 280,410 particles. An initial τA map was generated by using particles that showed high-resolution features in 2D classification. In addition, another set of particles in 2D classes with high resolution features were used for a second round of neural network training and particle picking in TOPAZ, which yielded 365,063 particles. Three rounds of heterogeneous refinement were performed to classify this final set of particles. The ab initio map of τA and three “junk maps,” previously generated in the first round of TOPAZ training, were used for the heterogeneous refinement step as input maps. A final nonuniform (NU) refinement applied to the best class from the last heterogeneous refinement, containing 55,079 particles, reached an overall resolution of 3.5 Å. For the τB-DNA subcomplex, a similar strategy was applied. After the first training step, 517,116 picked particles were subjected to 2D classification. The particles that belong to classes with high-resolution features were used for a subsequent TOPAZ training, while bad 2D classes were used to generate junk maps. A second round of TOPAZ training and picking generated 762,590 particles. A 2D classification step was applied, from which an ab initio τB-DNA map was generated and used for a heterogeneous refinement in the following step. A total of 206,812 particles were further sorted by 2D classification and ab initio reconstruction (three classes). NU refinement was applied to one class with 99,217 particles reaching an overall resolution of 3.3 Å. To further classify these particles, the continuous reconstruction program cryoDRGN (*56*) was used. The particles were binned to a box size of 256 × 256 pixels. A network with an architecture of three hidden layers with 512 neurons per layer for encoder and decoder and latent space size of 8 was trained for 50 epochs. The resulting clusters averages were mapped back to derive cryo-EM density maps. On the basis of visual inspection of these maps, a set of 35,379 particles was selected and subjected to NU refinement in cryoSPARC. In addition, a local refinement of each monomer in the τB-DNA map was performed yielding in both cases a 3.2-Å cryo-EM map. The final maps were postprocessed using DeepEMhancer (*57*).

### TFIIIC model building, refinement, and analysis

Coot (*58*) was used for building of structural models. AlphaFold (*59*) predicted structures of all the hTFIIIC subunits were retrieved from the AlphaFold Protein Structure Database: TFIIIC220 (identifier: AF-Q12789-F1), TFIIIC110 (identifier: AF-Q8WUA4-F1), TFIIIC102 (identifier: AF-Q9Y5Q9-F1), TFIIIC90 (identifier: AF-Q9UKN8-F1), TFIIIC63 (identifier: AF-Q9Y5Q8-F1), and TFIIIC35 (identifier: AF-Q969F1-F1) and fitted into the density maps using ChimeraX (*60*). A B-DNA model was placed into the density with self-restraints. AlphaFold-multimer (*61*) was used to assess regions with low resolution. The models were iteratively subjected to real-space refinement in PHENIX (*62*) and manual adjustment in Coot. Validation of the refined model was performed using MolProbity (*63*). Surface interaction area was calculated by Proteins, Interfaces, Structures and Assemblies (PISA) (*64*). Protein-DNA interactions were analyzed by NUCPLOT (*65*). For τA, model building was restricted to the cryo-EM density derived from the sample in which TFIIIC was reconstituted with DNA. 3D classification and refinement of τA particles from the DNA unbound sample yielded a lower resolution cryo-EM density at 3.8 Å and indicated a preferred orientation of the particles toward the air-water interface (table S2). Rigid-body fitting of the model of τA from the DNA-bound sample into the cryo-EM density of τA from the DNA-unbound sample indicated no major conformational changes. Structural homologs of TFIIIC220 domains were retrieved from the Protein Data Bank database using the DALI (*66*) server. The DNA geometry was analyzed via Curves+ (*67*).

### Cryo-EM mapping of particle pair distances at a single-molecule level

The particle .star files of the final RELION 3D refinement of τA and the NU refinement of τB-dimer subcomplexes derived from the DNA-bound sample served as input data for the particle distance pair analysis. The names and refined coordinates of the τB-dimer complex and τA were extracted from each micrograph and organized into a distance matrix of size N_τB_ × N_τA_. The particles were paired with a complementing partner by determining the smallest value in the distance matrix, saving the corresponding particles, and deleting them from the matrix to avoid multiple pairings of a particle. These steps were repeated until all particles in the rows or columns are assigned. Then, a new distance matrix is calculated for the next micrograph. Thus, the algorithm iterates through all micrographs. The distances of all pairings in all micrographs were listed and plotted in a histogram (fig. S12A).

To estimate the number of linked and randomly paired particles, the distance of random particles was calculated. The same particle sets from the micrographs were used however with randomized coordinates. The simulated particle distances were obtained equally and compared to the measured distances of the particles (fig. S12A).

### B-box DNA sequence conservation

To investigate B-box DNA sequence conservation across eukaryotes, we downloaded a set of eukaryotic tRNA genes from the GtRNAdb database (Release 15, http://gtrnadb2009.ucsc.edu/) (*68*). For the analysis of human tRNA genes, we used the hg38 - GRCh38 dataset retrieved from the GtRNAdb 2.0 database (Release 19, http://gtrnadb.ucsc.edu/) genes (*69*). tRNA genes with a score below 70.0 were discarded for the sequence conservation analysis across eukaryotic tRNA genes, resulting in 35,829 genes. The tRNA genes were aligned using the “cmalign” program within the Inferal package (*70*) and the tRNA covariance model (RF00005), retrieved from the Rfam database (*71*). Given that the number of input genes exceeded the maximum number of input sequences for Infernal, the data were split, aligned separately with Infernal as four separate profiles (maximum 10,000 sequences, each), and afterward combined using the profile alignment function in MUSCLE (*72*). The alignment was manually trimmed to cover only the B-box DNA motif and adjusted and trimmed to remove column gaps, for which also 15 outliers had to be removed. The final alignment for the B-box DNA motif contained 35,814 tRNA genes from 65 eukaryotic species covering three eukaryotic supergroups. Sequence logos were generated via WebLogo (*73*).

### Analysis of TFIIIC220 sequence conservation

TFIIIC220 orthologs were obtained from the National Center for Biotechnology Information reference sequence database (*74*) via DELTA-BLAST (*75*) using human TFIIIC220 as query sequence. The retrieved protein sequence hits were filtered to reduce the bias on found opisthokont orthologs and, at the same time, cover a broad range of eukaryotic species (metazoan, 29; fungi, 29; amoebozoa, 1; plants, 59; discoba, 2). A multiple sequence alignment (MSA) was generated with MAFFT (*76*) and subjected to the residue sequence conservation analysis via Scorecons (*77*), for which the entropic (seven types) scoring method was used. MSA sections were selected and visualized with TeXshade (*78*). The phylogenetic tree of the TFIIIC220 WH1 to WH4 MSA was computed with FastTree 2 (*79*).
