# Supplementary material for: Structural insights into human TFIIIC promoter recognition
Source: Sci Adv. 2023 Jul 7;9(27):eadh2019. doi: 10.1126/sciadv.adh2019 (PMC11811891; doi:10.1126/sciadv.adh2019)
Supplement: Supplementary file 1 — Figs. S1 to S12 Tables S1 to S3 [file sciadv.adh2019_sm.pdf]

Science Advances
AAAS

Supplementary Materials for

Structural insights into human TFIIIC promoter recognition

Wolfram Seifert-Davila *et al.*

Corresponding author: Christoph W. Müller, christoph.mueller@embl.de

Sci. Adv. **9**, eadh2019 (2023)
DOI: 10.1126/sciadv.adh2019

This PDF file includes:

Figs. S1 to S12
Tables S1 to S3

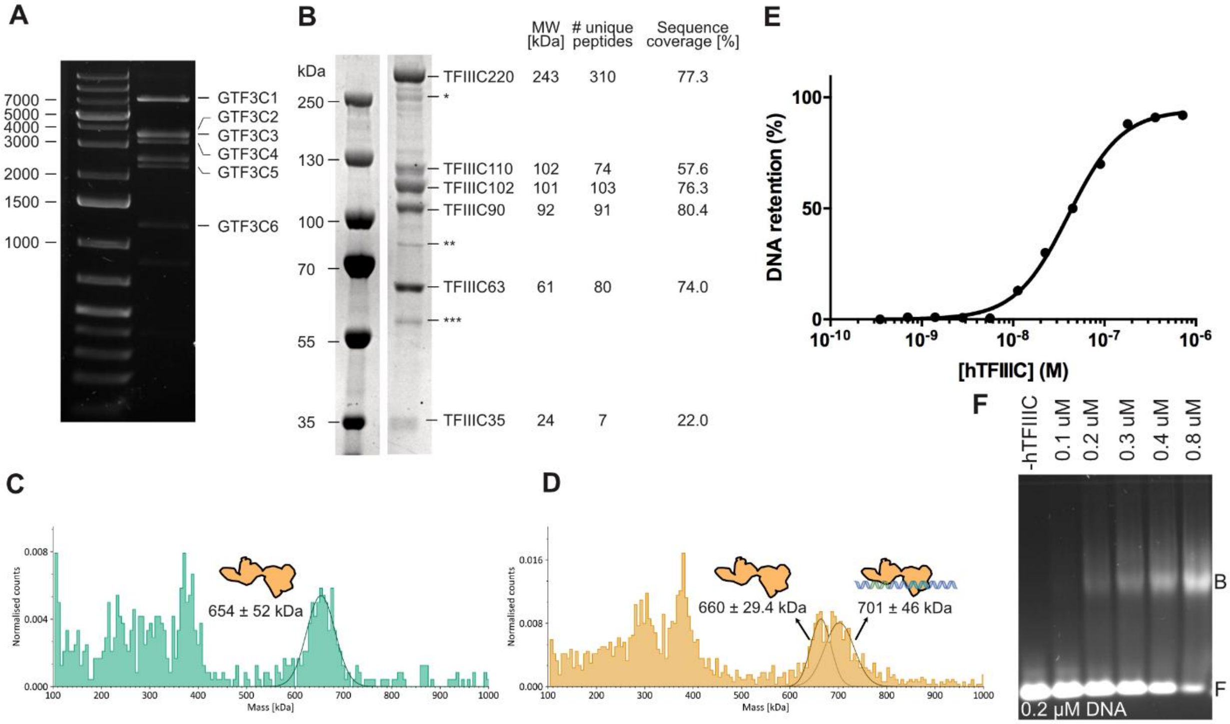

Fig. S1. Cloning, purification of hTFIIIC and analysis of hTFIIIC-DNA interaction.
(A) Analysis of the pBIG2ab vector containing all the hTFIIIC genes by restriction digest (SwaI) and gel electrophoresis. **(B)** SDS-PAGE of the purified hTFIIIC complex. All the subunits were identified by mass spectrometry. (*) indicates TFIIIC220 degradation products, (**) shows the presence of 70 kDa heat shock protein from the expression host, and (***) shows α- and β-tubulins from the host. **(C)** Mass photometry analysis of hTFIIIC. The mass distribution histogram is shown together with a schematic representation of hTFIIIC next to the peak with a molecular weight of 654 ± 52 kDa. **(D)** Mass photometry analysis of DNA-bound hTFIIIC. Mass distribution histogram of a sample containing hTFIIIC and DNA. A schematic presentation of hTFIIIC unbound (left) and bound (right) to DNA is shown next to the peaks corresponding to 660 ± 29.4 kDa and 701 ± 46 kDa, respectively. **(E)** Filter binding assay. hTFIIIC was titrated against radioactive labeled *TRR-TCT3-2* gene. The binding data was fitted to a Hill equation with a fixed Hill coefficient of 1. The K_D value was estimated as 40 nM (standard error: 2 nM). The standard error represents the uncertainty associated with our parameter estimate. N = 1. **(F)** EMSA demonstrates a concentration-dependent shift in DNA migration in presence of hTFIIIC. Notably, the ligand concentration used in this assay (0.2 μM) is well above the estimated K_D, and therefore, these conditions are not suitable for K_D estimation. The observed DNA bands are labeled as follows: F, free DNA; B, DNA bound to hTFIIIC. N = 1.

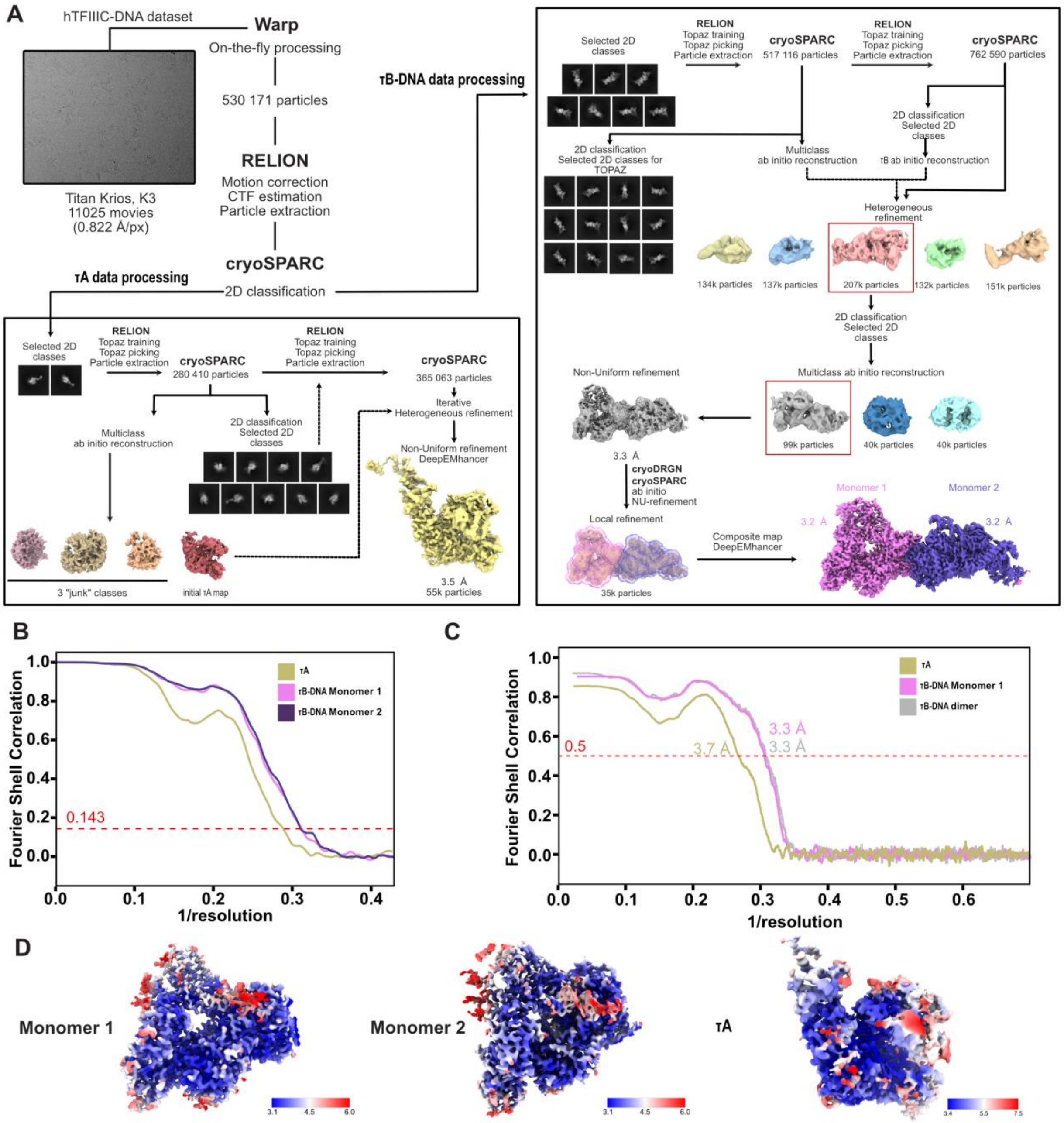

Fig. S2. Cryo-EM data processing and quality assessment of hTFIIIC.

(A) Processing pipeline of the hTFIIIC-DNA Titan Krios dataset. A representative micrograph, 2D classes, 3D classes from heterogeneous refinement and NU-refinement maps of τA (bottom left) and τB-DNA (right) from CryoSPARC, post-processed DeepEMhancer maps are shown. **(B)** FSC curves of the τA, monomer 1 and monomer 2 from the dimeric τB-DNA map show a final resolution of 3.5 Å, 3.2 Å and 3.2 Å, respectively (FSC = 0.143). **(C)** FSC between the map and the model to assess map-to-model fit (FSC = 0.5). **(D)** Local resolution estimation of τA, monomer 1 and monomer 2 implemented in RELION.

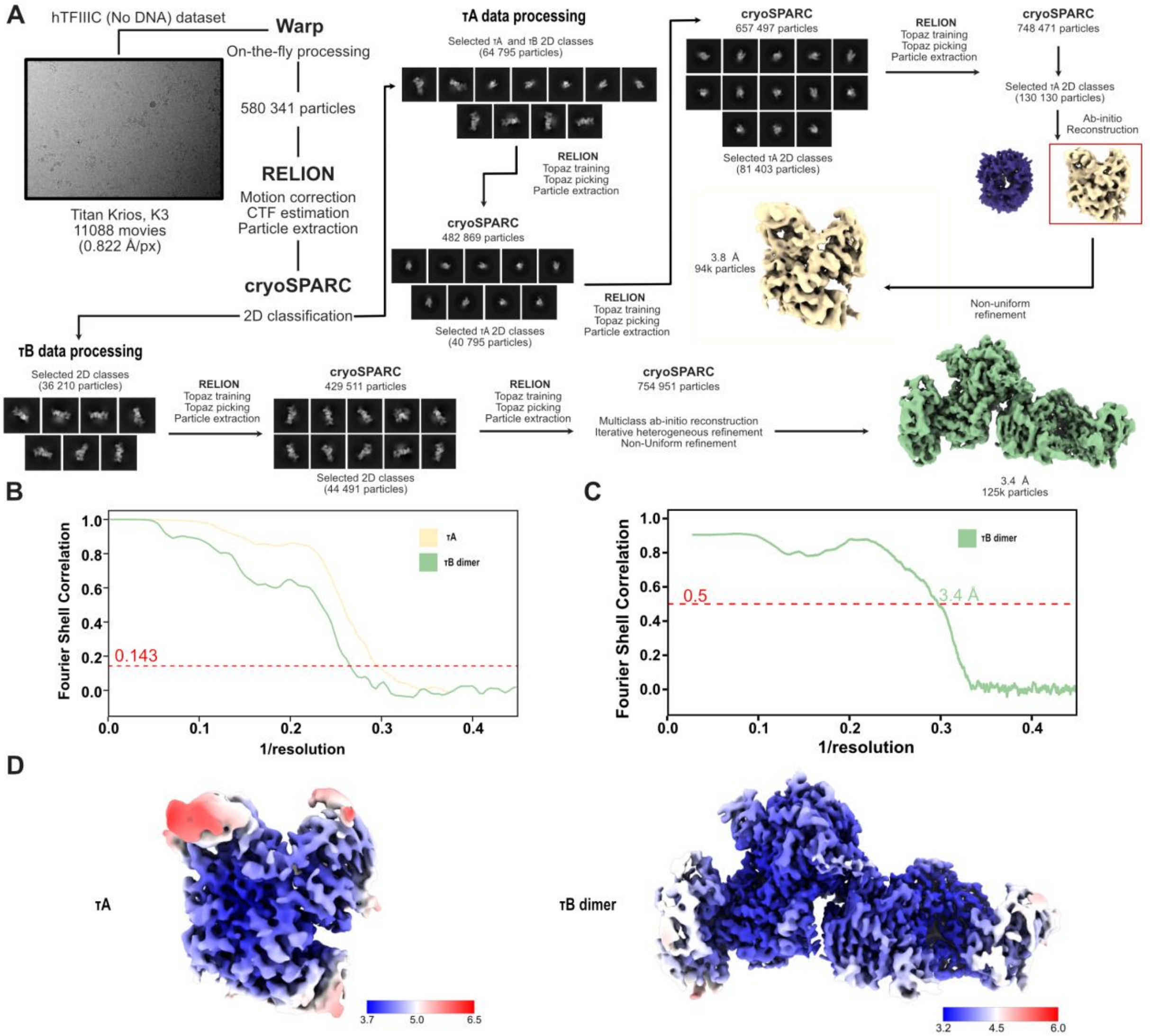

Fig. S3. Cryo-EM data processing and quality assessment of hTFIIIC (No DNA) sample

(A) Processing pipeline of the hTFIIIC (No DNA sample) Titan Krios dataset. A representative micrograph, 2D classes, 3D classes from ab-initio reconstruction and NU-refinement maps of τA (top right) and τB-DNA (right) from CryoSPARC. **(B)** FSC curves of the τA and dimeric τB map show a final resolution of 3.8 Å and 3.4 Å, respectively (FSC = 0.143). **(C)** FSC between the dimeric τB map and the model to assess map-to-model fit (FSC = 0.5). **(D)** Local resolution estimation of τA and the dimeric τB map implemented in RELION.

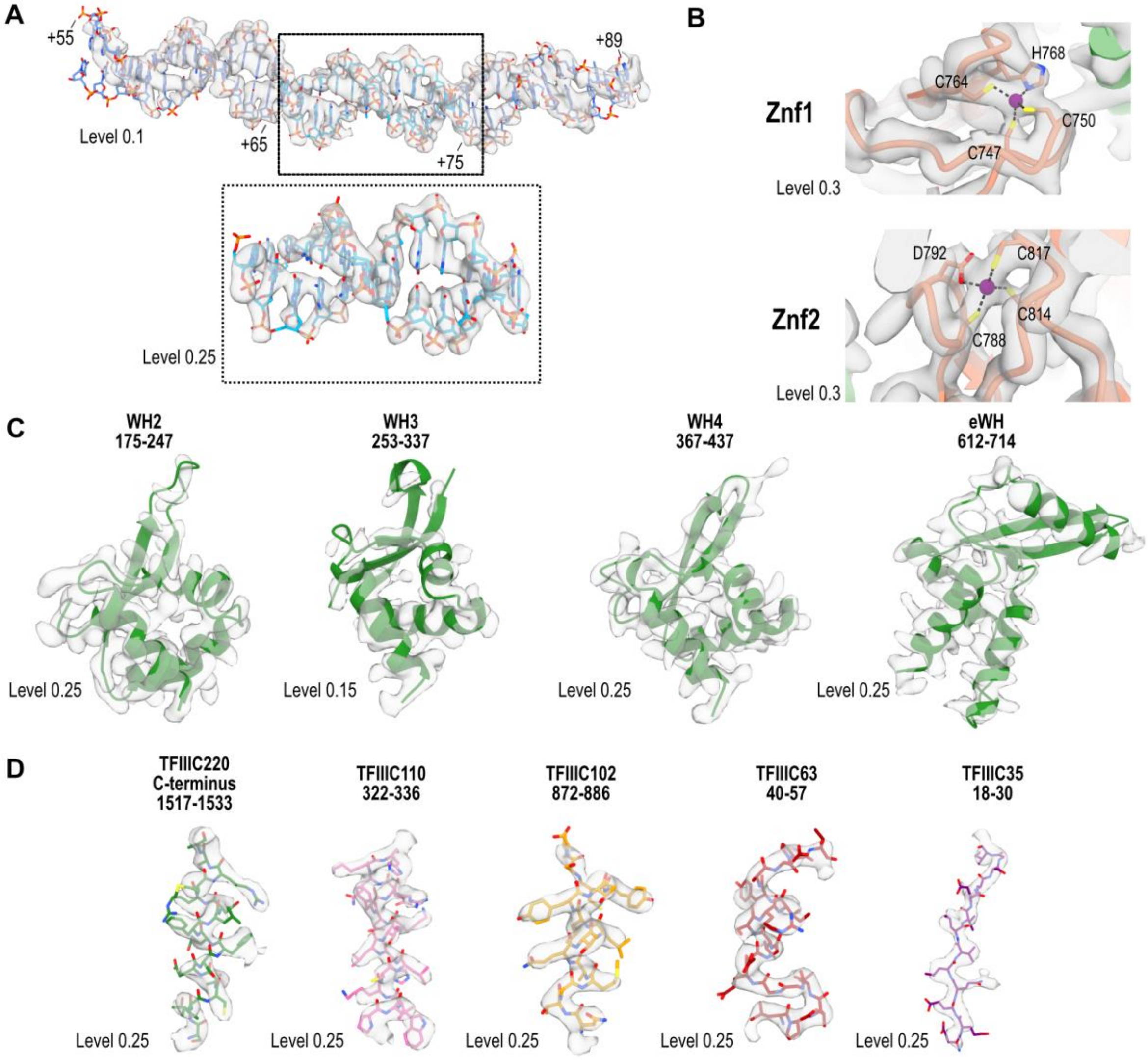

Fig. S4. Quality of map densities. Exemplary cryo-EM densities and refined models of: **(A)** DNA, including the B box (bottom panel), **(B)** Znf1 and Znf2, **(C)** WH2-4 and eWH, **(D)** TFIIIC220 C-terminus, TFIIIC110, TFIIIC102, TFIIIC90, TFIIIC63, TFIIIC35. The threshold level is indicated next to the model.

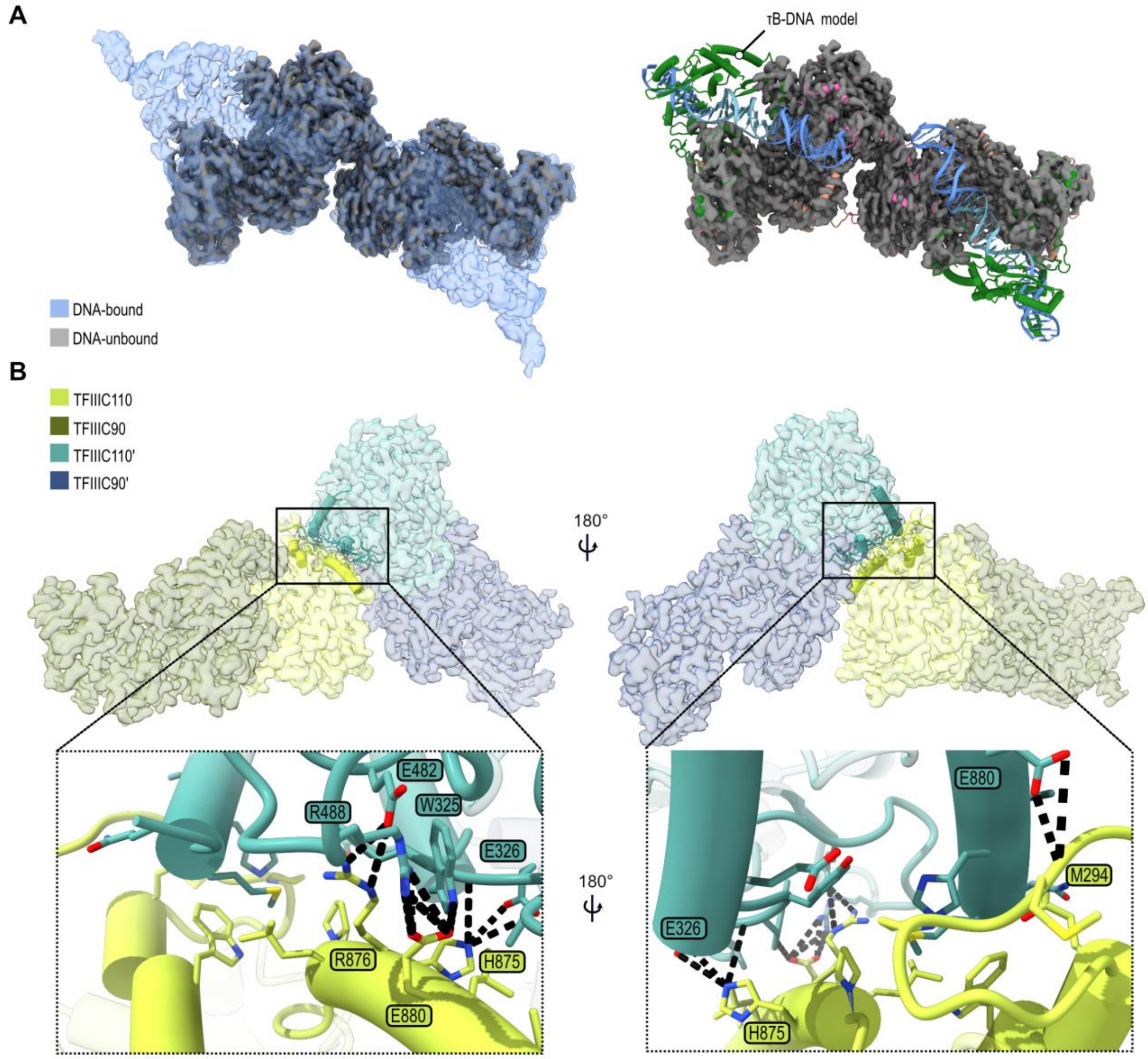

Fig. S5. Analysis of τB dimerization. (A) Left, Cryo-EM density map of DNA-unbound hTFIIIC (grey), obtained from a separate dataset without DNA, superimposed with DNA-bound TFIIIC map (blue and transparent). Right, superimposition of DNA-unbound TFIIIC map with the refined DNA-bound TFIIIC model, represented as cartoon. **(B)** Interface interaction between τB monomers. Insets show the amino acids, which take part in the interaction. Black dashed lines represent polar interactions with the amino acids in coloured boxes. The amino acids participating in the interactions were identified by ChimeraX "interfaces" command line and PDBePISA.

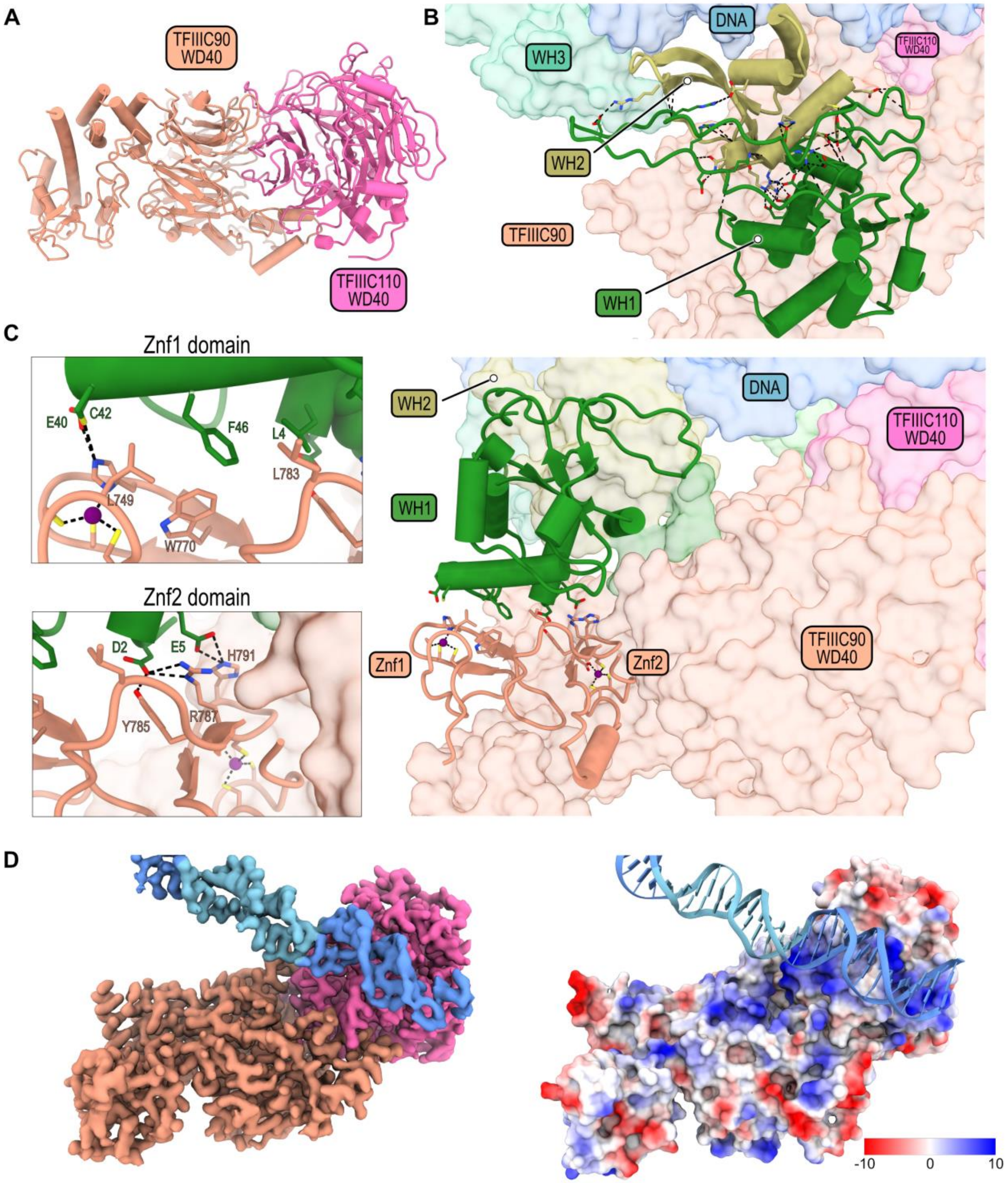

Fig. S6. τB core is important for complex stability and DNA interaction.
(A) Structural model of the τB core formed by the interaction of two WD40 domains. **(B)** Interaction of WH1 and WH2 domains. The sidechains of the amino acids participating in hydrogen bonds formation are shown. Hydrogen bonds are represented as black dashed lines. **(C)** Zinc fingers (Znf) 1 and 2 (cartoon representation) of the TFIIIC90 subunit interact with WH1 from the TFIIIC220 subunit. Insets show the chemical environment of this interaction. **(D)** Left, DeepEMhancer maps of the τB core and DNA (B-box in light blue). Right, electrostatic

(Coulomb) potential surface representation of the τB core showing a positively charged surface of the TFIIIC110-WD40 domain interacting with the DNA downstream of the B-box.

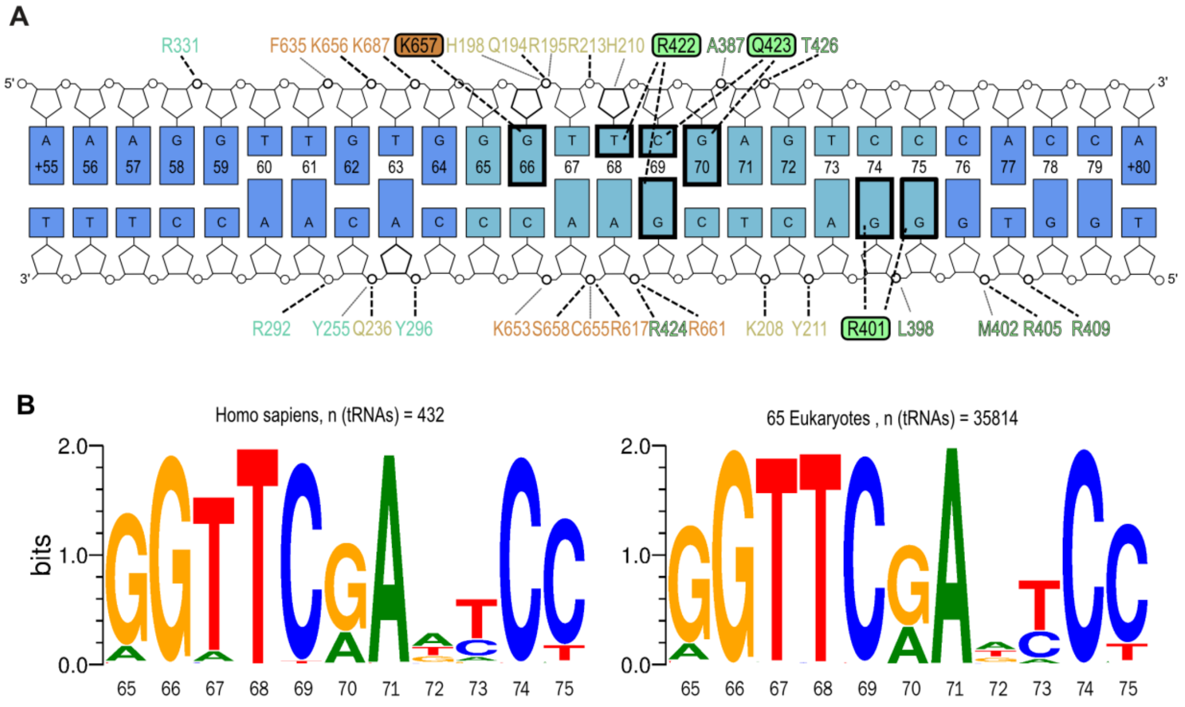

Fig. S7. DNA recognition by TFIIIC220 and analysis of B-box DNA sequence conservation. **(A)** Schematic of all protein-DNA interactions observed in the cryo-EM structure. Highlighted bases form H-bonds with the amino acids in coloured boxes. Domain-specific amino acid colours are the same as in Fig 2. H-bonds are depicted as bold, dashed lines and apolar contacts are shown as grey, thin lines. **(B)** B-box DNA sequence conservation across tRNA genes from human (left panel) and 65 eukaryotic species covering metazoa (44 taxa), fungi (11), plantaea (9), and kinetoplastida (1) (right panel). Sequence logos were computed via WebLogo (73). The human *TRR-TCT3-2* tRNA gene was used as a reference for the numbering of the nucleotide positions (shown below the sequence logos).

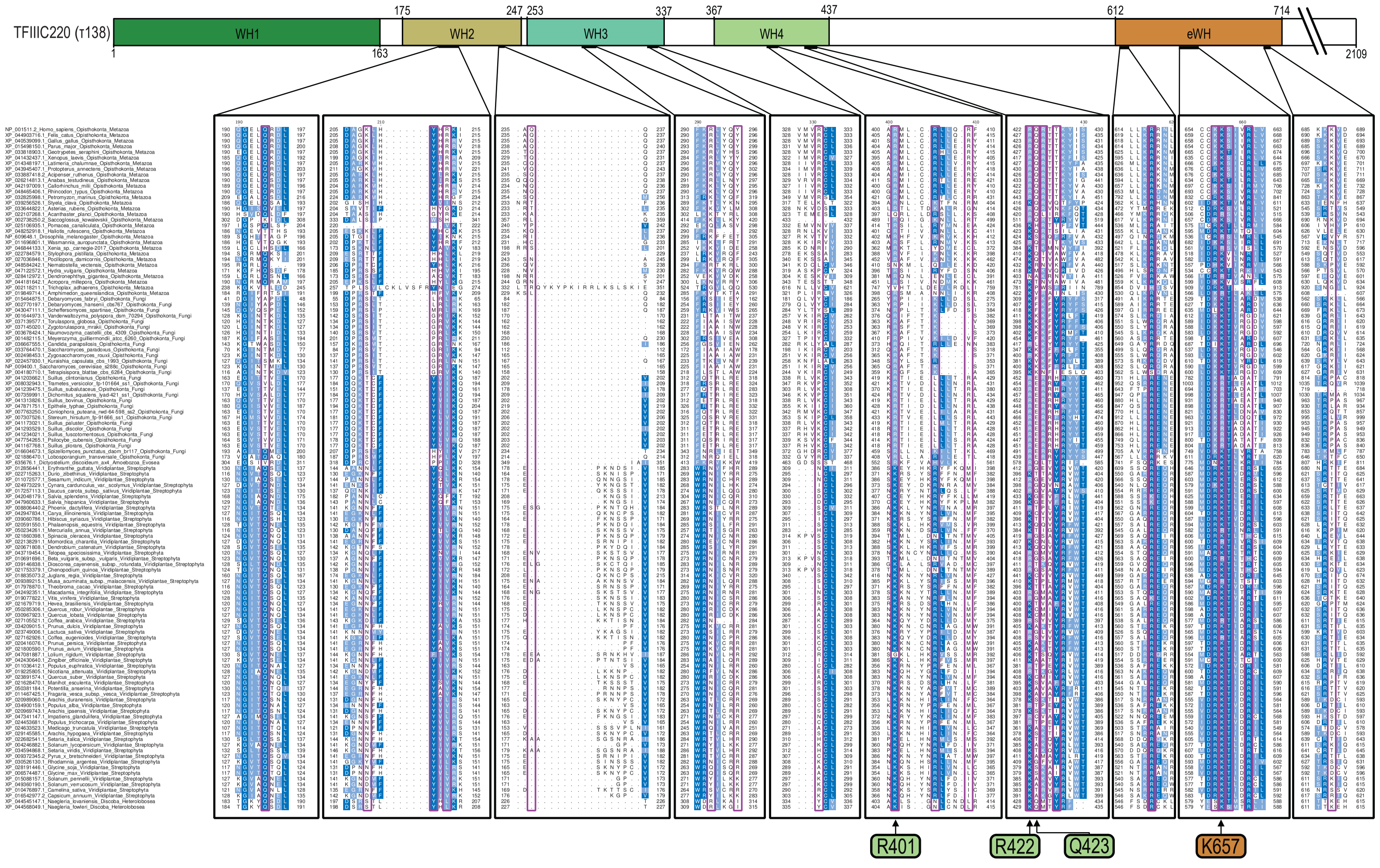
TFIIIC220 (τ138)
WH1
WH2
WH3
WH4
eWH
1
163
175
247
253
337
367
437
612
714
2109
R401
R422
Q423
K657

Fig. S8. Multiple sequence alignment (MSA) of TFIIIC220 orthologs. Top: schematic of the N-terminal half of human TFIIIC220, which was used as query to infer homologous proteins via DELTA-BLAST. Bottom: Selected sections of the MSA featuring residues that form polar contacts with the tDNA (marked with purple frames). Residues that form base-specific polar contacts are further highlighted on the bottom of the MSA. Positions of the selected sections are marked in the TFIIIC220 schematic.

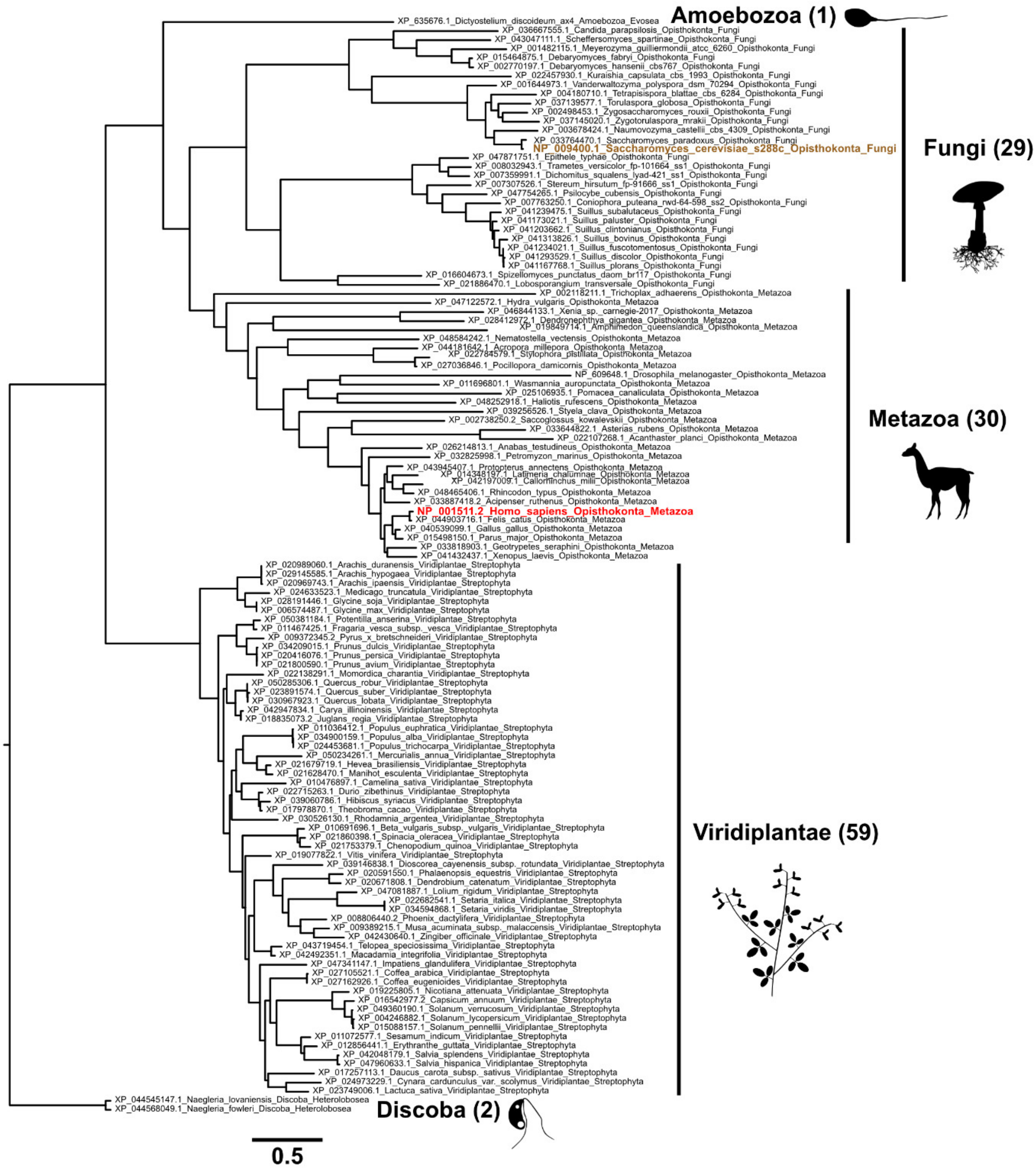

Fig. S9. Phylogenetic tree of the TFIIIC220 WH1-4-domains. The tree illustrates taxon sampling for the sequence conversation analysis of DNA-binding residues. The tree was calculated with FastTree 2.1 based on an input MSA of the TFIIIC220 WH1-4 domains, extracted from the MSA used for the ScoreCons analysis. The numbers of used taxa are given in parentheses. Scale bar – protein substitutions per site. Silhouettes were downloaded from https://www.phylopic.org. Attribution and license information is detailed under the following permalink:

https://www.phylopic.org/permalinks/12d59ab31f417829fa60120294bea4c813069710fc28093097e15b758cfc775f

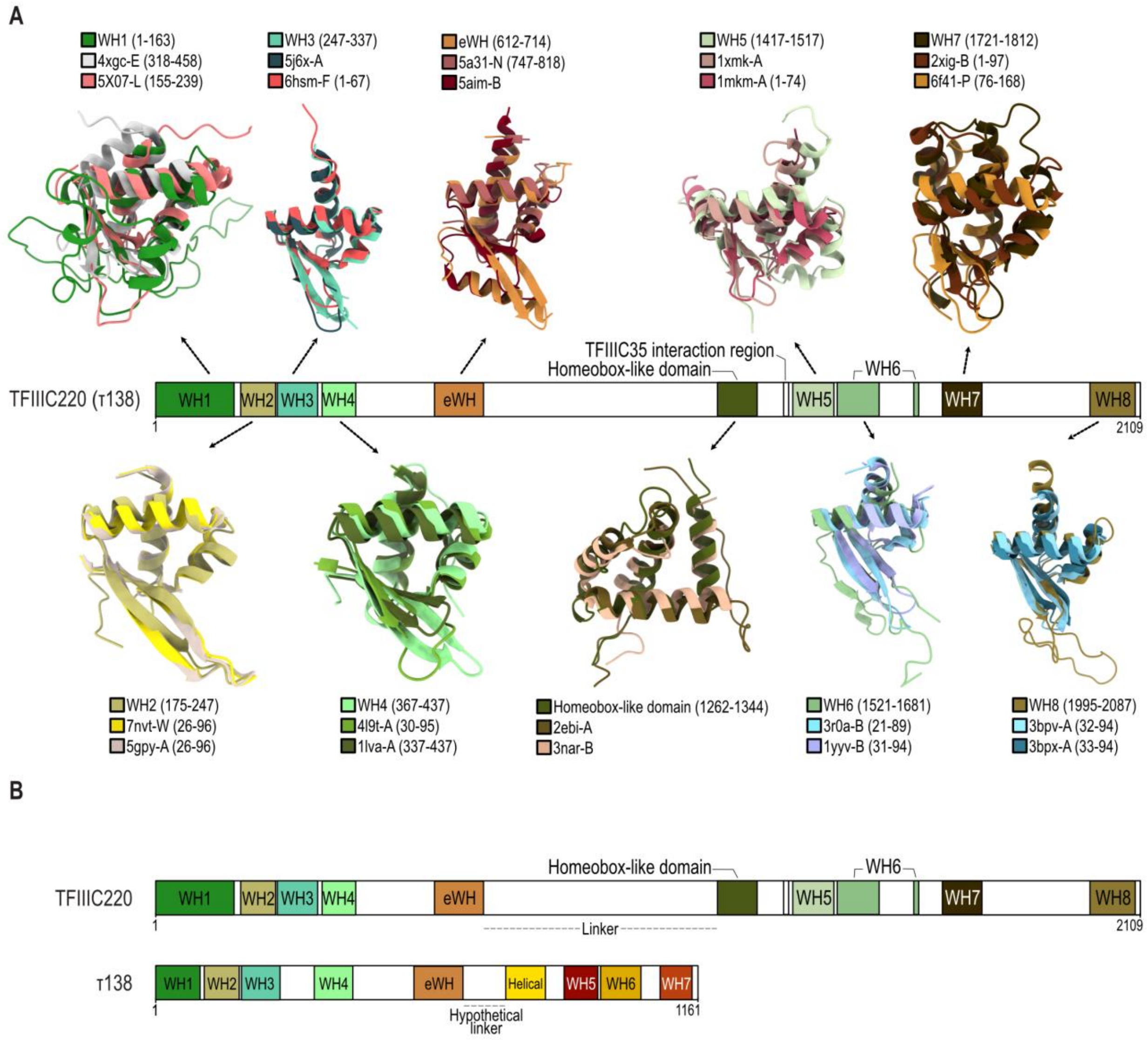

Fig. S10. TFIIIC220 subunit is mostly composed of winged-helix domains. (A) Superimposition of the structural models of the TFIIIC220 domains and two of their closest structural homologues retrieved from the Protein Data Bank database using the DALI server. The PDB IDs are written above (top models) and below (bottom models) each superimposition. The amino acid range for each domain is indicated in brackets. The colors of the TFIIIC220 domains are the same as in the domain architecture (middle). **(B)** Comparison of the domain architecture of TFIIIC220 (human) and τ138 (yeast). Domains that are structurally homologous between species are colored the same. The τ138 domain annotation was produced using the predicted τ138 Alphafold structure (ID: C7GTB1). The putative domain boundaries were determined using ChimeraX and the predicted aligned error (PAE).

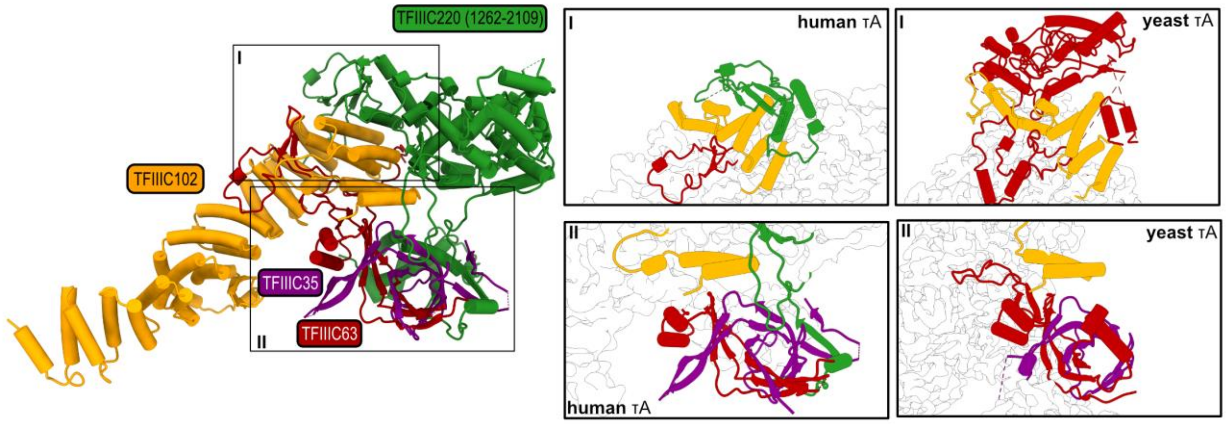

Fig. S11. Comparison between human τA and yeast τA. Overview of the interaction between TFIIIC102, TFIIIC63 and TFIIIC35 with the TFIIIC220 C-terminal moeity. (I) TFIIIC102 C-terminus TPR domain interacts with WH6 TFIIIC220 in human (left), while in yeast the τ131 C-terminus TPR domain interacts with the DNA-binding domain (DBD) of τ95 (right). (II) The WH8 and the TFIIIC35 IR of TFIIIC220 interact with TFIIICC63-TFIIIC35 dimer (left), absent in the yeast τA (right) (PDB ID: 6YJ6).

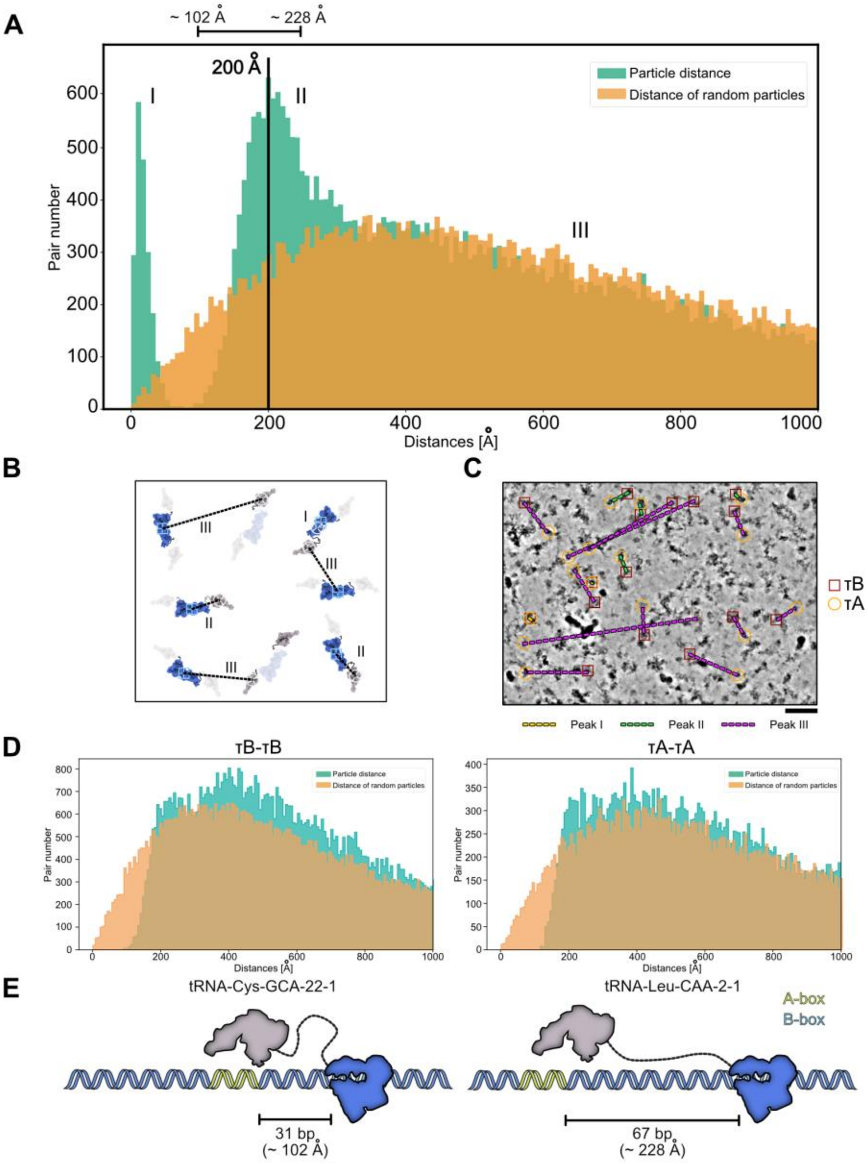

Fig. S12. τA and τB are connected by a flexible linker. (A) Histogram showing the distribution of distances between τA and τB-dimer in the micrograph (particle distance) versus the distance of randomly generated particles as control**.** The minimum and maximum distance between A and B box found in human tRNA gene is depicted above the histogram**. (B)** Schematics that illustrate particle distances representing three different cases: (I) mislabeled particles, (II) 200 Å distance between τA and τB-dimer within the same TFIIIC molecule, (III) distance between τA and τB-dimer from two different TFIIIC complexes. **(C)** An actual denoised micrograph showing τA and τB particles used in this analysis. The distances corresponding to different peaks are represented using distinct colors: Peak I (yellow): 1-100 Å, peak II (green): 101-280 Å, peak III (magenta): >281 Å. Scale bar: 500 Å. **(D)** Histograms showing the distance of τB-τB (left) and τA-τA (right) particles in comparison with randomly generated particles (control). **(E)** Schematics representing the flexibility of TFIIIC to bridge the smallest (*tRNA-Cys-GCA-22-1*) and largest (*tRNA-Leu-CAA-2-1)* distances between the A- and B-box present in the human tRNAs. τB is depicted in blue and τA in grey.

Table S1. Cryo-EM data collection, refinement and validation statistics

	τB-DNA<br>DNA sample<br>Monomer 1 map<br>(EMD-16713)<br>(PDB 8CLI)	τB-DNA dimer map<br>DNA sample<br>Composite map<br>(EMD-16714, EMD-17446, EMD-17447)<br>(PDB 8CLJ)	τA map<br>DNA sample<br>(EMD-16715)<br>(PDB 8CLK)	τB dimer map<br>No DNA sample<br>(EMD-16717)<br>(PDB 8CLL)
**Data collection and processing**				
Magnification	105,000	105,000	105,000	105,00
Voltage (kV)	300	300	300	300
Electron exposure (e–/Å^2)	42.8	42.8	42.8	39.6
Defocus range (μm)	0.7-1.7	0.7-1.7	0.7-1.7	0.7-1.7
Pixel size (Å)	0.822	0.822	0.822	0.822
Symmetry imposed	C1	C1	C1	C1
Initial particle images (no.)	762,590	762,590	365,063	754,951
Final particle images (no.)	35,379	35,379	55,079	125,600
Map resolution (Å)	3.2	3.2	3.5	3.4
FSC threshold	0.143	0.143	0.143	0.143
Map resolution range (Å)	3.1-9.9	n/a	3.4-13.4	3.2-10.9
**Refinement**				
Initial model used (Alphafold structure prediction)	AF-Q12789-F1, AF-Q8WUA4-F1, AF-Q9UKN8-F1	AF-Q12789-F1, AF-Q8WUA4-F1, AF-Q9UKN8-F1	AF-Q9Y5Q9-F1, AF-Q9Y5Q8-F1, AF-Q969F1-F1	AF-Q12789-F1, AF-Q8WUA4-F1, AF-Q9UKN8-F1
Model resolution (Å)	3.3	3.3	3.7	3.4
FSC threshold	0.5	0.5	0.5	0.5
Map sharpening *B* factor (Å^2)	-75.9	-	-114.5	-110.6
**Model composition**				
Non-hydrogen atoms	15955	31912	10800	25288
Protein / Nucleotide residues	1810 / 70	3620 / 140	1344 / 0	3152 / 0
Ligands	2x Zn	4x Zn	-	4x Zn
***B* factors (Å^2)**				
Protein (min/max/mean)	22.5/314.4 /95.5	12.4/303.3 /93.0	5.1/259.8 /117.6	13.0/306.9/115.1
Ligand (min/max/mean)	148.7/165.1/156.9	125.9/167.6/149.3	-	188.7/250.6/222.1
Nucleotide (min/max/mean)	5.9/207.1/104.1	2.4/301.9 /113.4	-	-
**R.m.s. deviations**				
Bond lengths (Å)	0.004	0.004	0.005	0.003
Bond angles (°)	0.716	0.753	0.754	0.662
**Validation**				
MolProbity score	1.13	1.11	1.31	1.03
Clashscore	2.62	2.74	4.17	2.24
Poor rotamers (%)	0.00	0.00	0.00	0.00
**Ramachandran plot**				
Favored (%)	97.6	97.8	97.4	97.9
Allowed (%)	2.4	2.2	2.6	2.1
Disallowed (%)	0.0	0.0	0.0	0.0
**Rama-Z score**				
Whole	-0.73(0.18)	-0.77(0.13)	-1.42(0.21)	-0.75(0.14)
Helix	-0.89(0.19)	-0.86(0.13)	-1.12(0.17)	-0.76(0.15)
Sheet	0.25(0.24)	0.23(0.17)	-0.30(0.45)	0.05(0.18)
Loop	-0.46(0.21)	-0.53(0.15)	-0.63(0.26)	-0.52(0.15)

Table S2. Cryo-EM data collection and refinement for τA without DNA

	τA map<br>No DNA sample<br>(EMD-16716)
**Data collection and processing**	
Magnification	105,00
Voltage (kV)	300
Electron exposure (e–/Å^2)	39.6
Defocus range (μm)	0.7-1.7
Pixel size (Å)	0.822
Symmetry imposed	C1
Initial particle images (no.)	748,471
Final particle images (no.)	94,103
Map resolution (Å)	3.8
FSC threshold	0.143
Map resolution range (Å)	3.7-14

Table S3. TFIIIC220 orthologs used for sequence conservation analysis. Protein sequences were inferred via DELTA-BLAST from the NCBI reference sequence database. Left: original FASTA protein headers (NCBI identifier, protein name, species name). Right: modified FASTA protein headers that include NCBI identifiers, species names, and taxonomic information.

Original FASTA protein headers	Modified FASTA protein headers
NP_001511.2 general transcription factor 3C polypeptide 1 isoform 1 [Homo sapiens]	NP_001511.2_Homo_sapiens_Opisthokonta_Metazoa
XP_044903716.1 general transcription factor 3C polypeptide 1 isoform X1 [Felis catus]	XP_044903716.1_Felis_catus_Opisthokonta_Metazoa
XP_040539099.1 general transcription factor 3C polypeptide 1 isoform X1 [Gallus gallus]	XP_040539099.1_Gallus_gallus_Opisthokonta_Metazoa
XP_015498150.1 general transcription factor 3C polypeptide 1 isoform X2 [Parus major]	XP_015498150.1_Parus_major_Opisthokonta_Metazoa
XP_033818903.1 general transcription factor 3C polypeptide 1 isoform X1 [Geotrypetes seraphini]	XP_033818903.1_Geotrypetes_seraphini_Opisthokonta_Metazoa
XP_041432437.1 general transcription factor 3C polypeptide 1 isoform X2 [Xenopus laevis]	XP_041432437.1_Xenopus_laevis_Opisthokonta_Metazoa
XP_014348197.1 PREDICTED: general transcription factor 3C polypeptide 1 [Latimeria chalumnae]	XP_014348197.1_Latimeria_chalumnae_Opisthokonta_Metazoa
XP_043945407.1 general transcription factor 3C polypeptide 1 [Protopterus annectens]	XP_043945407.1_Protopterus_annectens_Opisthokonta_Metazoa
XP_033887418.2 general transcription factor 3C polypeptide 1 isoform X1 [Acipenser ruthenus]	XP_033887418.2_Acipenser_ruthenus_Opisthokonta_Metazoa
XP_026214813.1 general transcription factor 3C polypeptide 1 [Anabas testudineus]	XP_026214813.1_Anabas_testudineus_Opisthokonta_Metazoa
XP_042197009.1 general transcription factor 3C polypeptide 1 [Callorhinchus milii]	XP_042197009.1_Callorhinchus_milii_Opisthokonta_Metazoa
XP_048465406.1 general transcription factor 3C polypeptide 1 isoform X2 [Rhincodon typus]	XP_048465406.1_Rhincodon_typus_Opisthokonta_Metazoa
XP_032825998.1 general transcription factor 3C polypeptide 1 isoform X2 [Petromyzon marinus]	XP_032825998.1_Petromyzon_marinus_Opisthokonta_Metazoa
XP_039256526.1 LOW QUALITY PROTEIN: general transcription factor 3C polypeptide 1-like [Styela clava]	XP_039256526.1_Styela_clava_Opisthokonta_Metazoa
XP_033644822.1 general transcription factor 3C polypeptide 1-like isoform X1 [Asterias rubens]	XP_033644822.1_Asterias_rubens_Opisthokonta_Metazoa
XP_022107268.1 general transcription factor 3C polypeptide 1-like [Acanthaster planci]	XP_022107268.1_Acanthaster_planci_Opisthokonta_Metazoa
XP_002738250.2 PREDICTED: general transcription factor 3C polypeptide 1-like [Saccoglossus kowalevskii]	XP_002738250.2_Saccoglossus_kowalevskii_Opisthokonta_Metazoa
XP_025106935.1 general transcription factor 3C polypeptide 1-like [Pomacea canaliculata]	XP_025106935.1_Pomacea_canaliculata_Opisthokonta_Metazoa
XP_048252918.1 general transcription factor 3C polypeptide 1-like isoform X2 [Haliotis rufescens]	XP_048252918.1_Haliotis_rufescens_Opisthokonta_Metazoa
NP_609648.1 uncharacterized protein Dmel_CG7099 [Drosophila melanogaster]	NP_609648.1_Drosophila_melanogaster_Opisthokonta_Metazoa
XP_011696801.1 PREDICTED: general transcription factor 3C polypeptide 1 isoform X2 [Wasmannia auropunctata]	XP_011696801.1_Wasmannia_auropunctata_Opisthokonta_Metazoa
XP_046844133.1 general transcription factor 3C polypeptide 1-like [Xenia sp. Carnegie-2017]	XP_046844133.1_Xenia_sp._carnegie-2017_Opisthokonta_Metazoa
XP_022784579.1 general transcription factor 3C polypeptide 1-like [Stylophora pistillata]	XP_022784579.1_Stylophora_pistillata_Opisthokonta_Metazoa
XP_027036846.1 general transcription factor 3C polypeptide 1-like [Pocillopora damicornis]	XP_027036846.1_Pocillopora_damicornis_Opisthokonta_Metazoa
XP_048584242.1 general transcription factor 3C polypeptide 1 [Nematostella vectensis]	XP_048584242.1_Nematostella_vectensis_Opisthokonta_Metazoa
XP_047122572.1 general transcription factor 3C polypeptide 1 [Hydra vulgaris]	XP_047122572.1_Hydra_vulgaris_Opisthokonta_Metazoa
XP_028412972.1 general transcription factor 3C polypeptide 1-like [Dendronephthya gigantea]	XP_028412972.1_Dendronephthya_gigantea_Opisthokonta_Metazoa
XP_044181642.1 general transcription factor 3C polypeptide 1-like isoform X6 [Acropora millepora]	XP_044181642.1_Acropora_millepora_Opisthokonta_Metazoa
XP_002118211.1 hypothetical protein TRIADDRAFT_62269 [Trichoplax adhaerens]	XP_002118211.1_Trichoplax_adhaerens_Opisthokonta_Metazoa
XP_019849714.1 PREDICTED: general transcription factor 3C polypeptide 1-like isoform X2 [Amphimedon queenslandica]	XP_019849714.1_Amphimedon_queenslandica_Opisthokonta_Metazoa
XP_015464875.1 uncharacterized protein AC631_05472 [Debaryomyces fabryi]	XP_015464875.1_Debaryomyces_fabryi_Opisthokonta_Fungi
XP_002770197.1 DEHA2C11484p [Debaryomyces hansenii CBS767]	XP_002770197.1_Debaryomyces_hansenii_cbs767_Opisthokonta_Fungi
XP_043047111.1 RNA polymerase III transcription initiation factor complex subunit [Scheffersomyces spartinae]	XP_043047111.1_Scheffersomyces_spartinae_Opisthokonta_Fungi
XP_001644973.1 hypothetical protein Kpol_1025p35 [Vanderwaltozyma polyspora DSM 70294]	XP_001644973.1_Vanderwaltozyma_polyspora_dsm_70294_Opisthokonta_Fungi
XP_037139577.1 uncharacterized protein HG536_0D04250 [Torulaspora globosa]	XP_037139577.1_Torulaspora_globosa_Opisthokonta_Fungi
XP_037145020.1 uncharacterized protein HG535_0E03770 [Zygotorulaspora mrakii]	XP_037145020.1_Zygotorulaspora_mrakii_Opisthokonta_Fungi
XP_003678424.1 hypothetical protein NCAS_0J01060 [Naumovozyma castellii CBS 4309]	XP_003678424.1_Naumovozyma_castellii_cbs_4309_Opisthokonta_Fungi
XP_001482115.1 hypothetical protein PGUG_05877 [Meyerozyma guilliermondii ATCC 6260]	XP_001482115.1_Meyerozyma_guilliermondii_atcc_6260_Opisthokonta_Fungi
XP_036667555.1 uncharacterized protein CPAR2_401940 [Candida parapsilosis]	XP_036667555.1_Candida_parapsilosis_Opisthokonta_Fungi
XP_033764470.1 Tfc3 [Saccharomyces paradoxus]	XP_033764470.1_Saccharomyces_paradoxus_Opisthokonta_Fungi
XP_002498453.1 uncharacterized protein ZYRO0G10648g [Zygosaccharomyces rouxii]	XP_002498453.1_Zygosaccharomyces_rouxii_Opisthokonta_Fungi
XP_022457930.1 uncharacterized protein KUCA_T00001891001 [Kuraishia capsulata CBS 1993]	XP_022457930.1_Kuraishia_capsulata_cbs_1993_Opisthokonta_Fungi
NP_009400.1 transcription factor TFIIIC subunit TFC3 [Saccharomyces cerevisiae S288C]	NP_009400.1_Saccharomyces_cerevisiae_s288c_Opisthokonta_Fungi
XP_004180710.1 hypothetical protein TBLA_0E01310 [Tetrapisispora blattae CBS 6284]	XP_004180710.1_Tetrapisispora_blattae_cbs_6284_Opisthokonta_Fungi
XP_041203662.1 uncharacterized protein DEU56DRAFT_45236 [Suillus clintonianus]	XP_041203662.1_Suillus_clintonianus_Opisthokonta_Fungi
XP_008032943.1 uncharacterized protein TRAVEDRAFT_139409 [Trametes versicolor FP-101664 SS1]	XP_008032943.1_Trametes_versicolor_fp-101664_ss1_Opisthokonta_Fungi
XP_041239475.1 uncharacterized protein DFJ58DRAFT_845058 [Suillus subalutaceus]	XP_041239475.1_Suillus_subalutaceus_Opisthokonta_Fungi
XP_007359991.1 uncharacterized protein DICSQDRAFT_94776 [Dichomitus squalens LYAD-421 SS1]	XP_007359991.1_Dichomitus_squalens_lyad-421_ss1_Opisthokonta_Fungi
XP_041313826.1 uncharacterized protein EDB93DRAFT_1324544 [Suillus bovinus]	XP_041313826.1_Suillus_bovinus_Opisthokonta_Fungi
XP_047871751.1 uncharacterized protein BXZ73DRAFT_107525 [Epithele typhae]	XP_047871751.1_Epithele_typhae_Opisthokonta_Fungi
XP_007763250.1 hypothetical protein CONPUDRAFT_86434 [Coniophora puteana RWD-64-598 SS2]	XP_007763250.1_Coniophora_puteana_rwd-64-598_ss2_Opisthokonta_Fungi
XP_007307526.1 hypothetical protein STEHIDRAFT_133320 [Stereum hirsutum FP-91666 SS1]	XP_007307526.1_Stereum_hirsutum_fp-91666_ss1_Opisthokonta_Fungi
XP_041173021.1 uncharacterized protein EDB91DRAFT_786767 [Suillus paluster]	XP_041173021.1_Suillus_paluster_Opisthokonta_Fungi
XP_041293529.1 uncharacterized protein F5147DRAFT_691378 [Suillus discolor]	XP_041293529.1_Suillus_discolor_Opisthokonta_Fungi
XP_041234021.1 uncharacterized protein F5891DRAFT_993073 [Suillus fuscotomentosus]	XP_041234021.1_Suillus_fuscotomentosus_Opisthokonta_Fungi
XP_047754265.1 Transcription factor tau subunit sfc3 [Psilocybe cubensis]	XP_047754265.1_Psilocybe_cubensis_Opisthokonta_Fungi
XP_041167768.1 uncharacterized protein HD556DRAFT_1428263 [Suillus plorans]	XP_041167768.1_Suillus_plorans_Opisthokonta_Fungi
XP_016604673.1 hypothetical protein, variant [Spizellomyces punctatus DAOM BR117]	XP_016604673.1_Spizellomyces_punctatus_daom_br117_Opisthokonta_Fungi
XP_021886470.1 hypothetical protein BCR41DRAFT_344088 [Lobosporangium transversale]	XP_021886470.1_Lobosporangium_transversale_Opisthokonta_Fungi
XP_635676.1 winged helix DNA-binding domain-containing protein [Dictyostelium discoideum AX4]	XP_635676.1_Dictyostelium_discoideum_ax4_Amoebozoa_Evosea
XP_012856441.1 PREDICTED: uncharacterized protein LOC105975760 [Erythranthe guttata]	XP_012856441.1_Erythranthe_guttata_Viridiplantae_Streptophyta
XP_022715263.1 uncharacterized protein LOC111274686 isoform X3 [Durio zibethinus]	XP_022715263.1_Durio_zibethinus_Viridiplantae_Streptophyta
XP_011072577.1 uncharacterized protein LOC105157796 isoform X1 [Sesamum indicum]	XP_011072577.1_Sesamum_indicum_Viridiplantae_Streptophyta
XP_024973229.1 uncharacterized protein LOC112511735 isoform X7 [Cynara cardunculus var. scolymus]	XP_024973229.1_Cynara_cardunculus_var._scolymus_Viridiplantae_Streptophyta
XP_017257113.1 PREDICTED: uncharacterized protein LOC108226632 [Daucus carota subsp. sativus]	XP_017257113.1_Daucus_carota_subsp._sativus_Viridiplantae_Streptophyta
XP_042048179.1 uncharacterized protein LOC121794186 isoform X2 [Salvia splendens]	XP_042048179.1_Salvia_splendens_Viridiplantae_Streptophyta
XP_047960633.1 uncharacterized protein LOC125205627 isoform X2 [Salvia hispanica]	XP_047960633.1_Salvia_hispanica_Viridiplantae_Streptophyta
XP_008806440.2 uncharacterized protein LOC103719131 isoform X2 [Phoenix dactylifera]	XP_008806440.2_Phoenix_dactylifera_Viridiplantae_Streptophyta
XP_042947834.1 uncharacterized protein LOC122280838 [Carya illinoinensis]	XP_042947834.1_Carya_illinoinensis_Viridiplantae_Streptophyta
XP_039060786.1 uncharacterized protein LOC120204817 isoform X5 [Hibiscus syriacus]	XP_039060786.1_Hibiscus_syriacus_Viridiplantae_Streptophyta
XP_020591550.1 uncharacterized protein LOC110032283 isoform X3 [Phalaenopsis equestris]	XP_020591550.1_Phalaenopsis_equestris_Viridiplantae_Streptophyta
XP_050234261.1 uncharacterized protein LOC126682580 [Mercurialis annua]	XP_050234261.1_Mercurialis_annua_Viridiplantae_Streptophyta
XP_021860398.1 uncharacterized protein LOC110799434 isoform X3 [Spinacia oleracea]	XP_021860398.1_Spinacia_oleracea_Viridiplantae_Streptophyta
XP_022138291.1 uncharacterized protein LOC111009503 [Momordica charantia]	XP_022138291.1_Momordica_charantia_Viridiplantae_Streptophyta
XP_020671808.1 uncharacterized protein LOC110091869 isoform X6 [Dendrobium catenatum]	XP_020671808.1_Dendrobium_catenatum_Viridiplantae_Streptophyta
XP_043719454.1 uncharacterized protein LOC122667263 [Telopea speciosissima]	XP_043719454.1_Telopea_speciosissima_Viridiplantae_Streptophyta
XP_010691696.1 uncharacterized protein LOC104904960 isoform X1 [Beta vulgaris subsp. vulgaris]	XP_010691696.1_Beta_vulgaris_subsp._vulgaris_Viridiplantae_Streptophyta
XP_039146838.1 uncharacterized protein LOC120284099 [Dioscorea cayenensis subsp. rotundata]	XP_039146838.1_Dioscorea_cayenensis_subsp._rotundata_Viridiplantae_Streptophyta
XP_021753379.1 uncharacterized protein LOC110718782 [Chenopodium quinoa]	XP_021753379.1_Chenopodium_quinoa_Viridiplantae_Streptophyta
XP_018835073.2 uncharacterized protein LOC109001988 isoform X2 [Juglans regia]	XP_018835073.2_Juglans_regia_Viridiplantae_Streptophyta
XP_009389215.1 PREDICTED: uncharacterized protein LOC103975842 isoform X1 [Musa acuminata subsp. malaccensis]	XP_009389215.1_Musa_acuminata_subsp._malaccensis_Viridiplantae_Streptophyta
XP_017978870.1 PREDICTED: uncharacterized protein LOC18596208 isoform X2 [Theobroma cacao]	XP_017978870.1_Theobroma_cacao_Viridiplantae_Streptophyta
XP_042492351.1 uncharacterized protein LOC122071928 [Macadamia integrifolia]	XP_042492351.1_Macadamia_integrifolia_Viridiplantae_Streptophyta
XP_019077822.1 PREDICTED: uncharacterized protein LOC100267761 isoform X2 [Vitis vinifera]	XP_019077822.1_Vitis_vinifera_Viridiplantae_Streptophyta
XP_021679719.1 uncharacterized protein LOC110664370 isoform X2 [Hevea brasiliensis]	XP_021679719.1_Hevea_brasiliensis_Viridiplantae_Streptophyta

Table S3 (continued).

Original FASTA protein headers	Modified FASTA protein headers
XP_050285306.1 uncharacterized protein LOC126724932 [Quercus robur]	XP_050285306.1_Quercus_robur_Viridiplantae_Streptophyta
XP_030967923.1 uncharacterized protein LOC115988491 [Quercus lobata]	XP_030967923.1_Quercus_lobata_Viridiplantae_Streptophyta
XP_027105521.1 uncharacterized protein LOC113726159 isoform X2 [Coffea arabica]	XP_027105521.1_Coffea_arabica_Viridiplantae_Streptophyta
XP_034209015.1 uncharacterized protein LOC117622457 isoform X2 [Prunus dulcis]	XP_034209015.1_Prunus_dulcis_Viridiplantae_Streptophyta
XP_023749006.1 uncharacterized protein LOC111897275 isoform X2 [Lactuca sativa]	XP_023749006.1_Lactuca_sativa_Viridiplantae_Streptophyta
XP_021800590.1 uncharacterized protein LOC110744864 [Prunus avium]	XP_021800590.1_Prunus_avium_Viridiplantae_Streptophyta
XP_047081887.1 uncharacterized protein LOC124692535 [Lolium rigidum]	XP_047081887.1_Lolium_rigidum_Viridiplantae_Streptophyta
XP_042430640.1 uncharacterized protein LOC122017169 [Zingiber officinale]	XP_042430640.1_Zingiber_officinale_Viridiplantae_Streptophyta
XP_011036412.1 PREDICTED: uncharacterized protein LOC105133943 isoform X3 [Populus euphratica]	XP_011036412.1_Populus_euphratica_Viridiplantae_Streptophyta
XP_019225805.1 PREDICTED: uncharacterized protein LOC109207356 isoform X1 [Nicotiana attenuata]	XP_019225805.1_Nicotiana_attenuata_Viridiplantae_Streptophyta
XP_023891574.1 uncharacterized protein LOC112003595 [Quercus suber]	XP_023891574.1_Quercus_suber_Viridiplantae_Streptophyta
XP_021628470.1 uncharacterized protein LOC110626726 isoform X2 [Manihot esculenta]	XP_021628470.1_Manihot_esculenta_Viridiplantae_Streptophyta
XP_050381184.1 uncharacterized protein LOC126798301 isoform X2 [Potentilla anserina]	XP_050381184.1_Potentilla_anserina_Viridiplantae_Streptophyta
XP_011467425.1 PREDICTED: uncharacterized protein LOC101308114 isoform X1 [Fragaria vesca subsp. vesca]	XP_011467425.1_Fragaria_vesca_subsp._vesca_Viridiplantae_Streptophyta
XP_020989060.1 uncharacterized protein LOC107470930 isoform X4 [Arachis duranensis]	XP_020989060.1_Arachis_duranensis_Viridiplantae_Streptophyta
XP_034900159.1 uncharacterized protein LOC118038023 isoform X3 [Populus alba]	XP_034900159.1_Populus_alba_Viridiplantae_Streptophyta
XP_020969743.1 uncharacterized protein LOC107622687 isoform X4 [Arachis ipaensis]	XP_020969743.1_Arachis_ipaensis_Viridiplantae_Streptophyta
XP_047341147.1 uncharacterized protein LOC124944847 [Impatiens glandulifera]	XP_047341147.1_Impatiens_glandulifera_Viridiplantae_Streptophyta
XP_024453681.1 uncharacterized protein LOC112326972 isoform X3 [Populus trichocarpa]	XP_024453681.1_Populus_trichocarpa_Viridiplantae_Streptophyta
XP_024633523.1 uncharacterized protein LOC11419389 isoform X3 [Medicago truncatula]	XP_024633523.1_Medicago_truncatula_Viridiplantae_Streptophyta
XP_029145585.1 uncharacterized protein LOC112716216 isoform X3 [Arachis hypogaea]	XP_029145585.1_Arachis_hypogaea_Viridiplantae_Streptophyta
XP_022682541.1 uncharacterized protein LOC101770976 isoform X3 [Setaria italica]	XP_022682541.1_Setaria_italica_Viridiplantae_Streptophyta
XP_004246882.1 uncharacterized protein LOC101258404 [Solanum lycopersicum]	XP_004246882.1_Solanum_lycopersicum_Viridiplantae_Streptophyta
XP_034594868.1 uncharacterized protein LOC117856633 isoform X6 [Setaria viridis]	XP_034594868.1_Setaria_viridis_Viridiplantae_Streptophyta
XP_009372345.2 uncharacterized protein LOC103961518 [Pyrus x bretschneideri]	XP_009372345.2_Pyrus_x_bretschneideri_Viridiplantae_Streptophyta
XP_030526130.1 uncharacterized protein LOC115737876 isoform X5 [Rhodamnia argentea]	XP_030526130.1_Rhodamnia_argentea_Viridiplantae_Streptophyta
XP_028191446.1 uncharacterized protein LOC114377194 isoform X5 [Glycine soja]	XP_028191446.1_Glycine_soja_Viridiplantae_Streptophyta
XP_006574487.1 uncharacterized protein LOC100814813 isoform X3 [Glycine max]	XP_006574487.1_Glycine_max_Viridiplantae_Streptophyta
XP_015088157.1 uncharacterized protein LOC107031346 [Solanum pennellii]	XP_015088157.1_Solanum_pennellii_Viridiplantae_Streptophyta
XP_049360190.1 uncharacterized protein LOC125824889 [Solanum verrucosum]	XP_049360190.1_Solanum_verrucosum_Viridiplantae_Streptophyta
XP_010476897.1 PREDICTED: uncharacterized protein LOC104756077 [Camelina sativa]	XP_010476897.1_Camelina_sativa_Viridiplantae_Streptophyta
XP_016542977.2 uncharacterized protein LOC107843239 isoform X1 [Capsicum annuum]	XP_016542977.2_Capsicum_annuum_Viridiplantae_Streptophyta
XP_044545147.1 uncharacterized protein C9374_008970 [Naegleria lovaniensis]	XP_044545147.1_Naegleria_lovaniensis_Discoba_Heterolobosea
XP_044568049.1 uncharacterized protein FDP41_010401 [Naegleria fowleri]	XP_044568049.1_Naegleria_fowleri_Discoba_Heterolobosea
